# Insulin-like growth factor binding protein 3 promotes radiosensitivity of oral squamous cell carcinoma cells via positive feedback on NF-κB/IL-6/ROS signaling

**DOI:** 10.1186/s13046-021-01898-7

**Published:** 2021-03-13

**Authors:** Ssu-Han Wang, Yu-Lin Chen, Jenn-Ren Hsiao, Fang-Yu Tsai, Shih Sheng Jiang, Alan Yueh-Luen Lee, Hui-Jen Tsai, Ya-Wen Chen

**Affiliations:** 1 National Institute of Cancer Research, National Health Research Institutes, 35, Keyan Road, Zhunan Town, Miaoli County, 35053 Taiwan; 2 Department of Otolaryngology, College of Medicine, National Cheng Kung University Hospital, National Cheng Kung University, Tainan, Taiwan; 3 National Institute of Cancer Research, National Health Research Institutes, Tainan, Taiwan; 4 Graduate Institute of Basic Medical Science, China Medical University, Taichung, Taiwan

**Keywords:** IGFBP3, Oral squamous cell carcinoma cells, Radiation, ROS, NF-κB

## Abstract

**Background:**

Ectopic insulin-like growth factor binding protein 3 (IGFBP3) expression has been shown to enhance cell migration and lymph node metastasis of oral squamous cell carcinoma (OSCC) cells. However, OSCC patients with high IGFBP3 expression had improved survival compared with those with low expression. Therefore, we speculated that IGFBP3 expression may play a role in response to conventional OSCC therapies, such as radiotherapy.

**Methods:**

We used in vitro and in vivo analyses to explore IGFBP3-mediated radiosensitivity. Reactive oxygen species (ROS) detection by flow cytometry was used to confirm IGFBP3-mediated ionizing radiation (IR)-induced apoptosis. Geneset enrichment analysis (GSEA) and ingenuity pathway analysis (IPA) were used to analyze the relationship between IGFBP3 and nuclear factor kappa-light-chain-enhancer of activated B cells (NF-κB) signaling. Assays involving an NF-κB inhibitor, ROS scavenger or interleukin 6 (IL-6) were used to evaluate the NF-κB/IL-6/ROS signaling in IGFBP3-mediated radiosensitivity.

**Results:**

Ectopic IGFBP3 expression enhanced IR-induced cell-killing in vitro. In vivo, IGFBP3 reduced tumor growth and increased apoptotic signals of tumor tissues in immunocompromised mice treated with IR. Combined with IR, ectopic IGFBP3 expression induced mitochondria-dependent apoptosis, which was apparent through mitochondrial destruction and increased ROS production. Ectopic IGFBP3 expression enhanced NK-κB activation and downstream cytokine expression. After IR exposure, IGFBP3-induced NF-κB activation was inhibited by the ROS scavenger N-acetyl-L-cysteine (NAC). IGFBP3-mediated ROS production was reduced by the NF-κB inhibitor BMS-345541, while exogenous IL-6 rescued the NF-κB-inhibited, IGFBP3-mediated ROS production.

**Conclusions:**

Our data demonstrate that IGFBP3, a potential biomarker for radiosensitivity, promotes IR-mediated OSCC cell death by increasing ROS production through NF-κB activation and cytokine production.

**Supplementary Information:**

The online version contains supplementary material available at 10.1186/s13046-021-01898-7.

## Background

Oral squamous cell carcinoma (OSCC) is one of the most common malignancies [1, 2]. Radiation therapy is a common adjuvant treatment for OSCC patients with or without prior surgery, and efficiently reduces tumor infiltration and growth while maintaining the integral morphology of the oral cavity and improving overall survival rates [3]. However, the radiosensitivity of individual tumors varies widely and radioresistance may contribute to OSCC treatment failure. Therefore, there is a need to identify biomarkers that predict radiosensitivity, treatment response and prognosis to provide targeted therapeutic strategies to patients based on their likelihood to respond to radiation therapy [4].

Ionizing radiation (IR) is one of the most effective cancer treatments due to its ability to directly induce DNA damage and indirectly generate reactive oxygen species (ROS). The direct and indirect effects of IR initiate a series of biological events that may repair the damage or culminate in permanent physiological changes or apoptosis [5]. Apoptosis, a tightly regulated and highly conserved process of cell death, can be triggered by intrinsic and extrinsic signaling pathways. ROS play a central role in regulation of apoptosis mediated by mitochondria, death receptors and the endoplasmic reticulum [6]. The mitochondrial pathway of apoptosis, and particularly mitochondrial outer membrane permeabilization, is regulated by proteins belonging to the B-cell-lymphoma protein 2 (Bcl-2) family [7]. In response to many types of stress or damage, mitochondrial outer membrane permeabilization releases pro-apoptotic proteins, such as cytochrome c into the cytosol. Studies have shown that opening of the mitochondrial permeability transition pore also induces depolarization of the transmembrane potential and loss of oxidative phosphorylation [8]. Through induction of apoptosis and other pathways, IR destroys mitochondrial functions, enhances mitochondrial oxidative stress, and leads to a production of mitochondrial ROS. Mitochondrial ROS are likely to act as signalling molecules for intracellular communication and may increase subsequent effects of radiation [9].

Insulin-like growth factor binding protein 3 (IGFBP3), a secretory glycoprotein, can modulate the mitogenic activity of insulin-like growth factor 1 receptor (IGF1R) [10]. IGF-independent role of IGFBP3 include its interactions with the extracellular, membrane and intracellular proteins and translocation into the cytoplasm and into the nucleus [11]. Abnormal expression or malfunction of IGFBP3 is associated with cancer development and progression. A series of studies have confirmed that IGFBP3 inhibits cell adhesion [12], invasiveness of endometrial cancer [13], metastasis in prostate cancer [14], and angiogenesis in head and neck squamous cell carcinoma [15]. Conversely, IGFBP3 has antioxidative activity, suppressing ROS [16], enhancing epithelial-to-mesenchymal transition and motility [17, 18], which is necessary for tumor progression. Thus, IGFBP3 may have context-dependent tumor-promoting or -suppressing activities. IGFBP3 has been shown to module radiosensitivity in different types of cancers. Ectopic IGFBP3 expression in p53-independent human breast cancer cells appeared to enhance radiosensitivity, with apoptosis induced through Bax and Bcl-2 after irradiation [19]. Esophageal squamous cell carcinoma cells with IGFBP3 knockdown had enhanced relative radioresistance [20]. Inversely, the enhanced expression of IGFBP3 in OSCC cells reduced radiosensitivity by activating DNA repair [21]. These studies suggest that IGFBP3 may be a key biomarker of cancer radiotherapy.

NF-κB proteins are a family of transcription factors that play important roles in inflammation and immunity [22]. Two main signaling pathways lead to the activation of NF-κB target genes. Of these, the canonical NF-κB pathway responds to diverse stimuli, including ligands of various cytokine receptors, pattern-recognition receptors, tumor necrosis factor receptor superfamily members, as well as both T-cell and B-cell receptors. The primary mechanism for canonical NF-κB activation is the inducible degradation of kappa light polypeptide gene enhancer in B-cells inhibitor, alpha (IκBα) triggered through its site-specific phosphorylation by a multi-subunit IκB kinase (IKK) complex. Upon activation, IKK phosphorylates IκBα at two N-terminal serine residues, triggering ubiquitin-dependent IκBα degradation in the proteasome and rapid and transient nuclear translocation of canonical NF-κB family members [23].

Different from the previous study [21], we found that OSCC patients with high levels of IGFBP3 had improved survival compared to those with low levels of IGFBP3. Ectopic IGFBP3 expression enhanced IR-induced cell-killing in vivo and in vitro. Under IR, IGFBP3-induced NF-κB activation was reduced by ROS inhibition. IGFBP3-mediated ROS production was disrupted by IKK inhibition, while exogenous IL-6 rescued the NF-κB-inhibited, IGFBP3-mediated ROS production. Our data suggest that IGFBP3 promotes IR-induced OSCC cell death via positive feedback regulation of ROS production by inducing NF-κB activation and cytokine expression.

## Methods

### Cell culture and chemicals

OEC-M1, TW2.6, and LN1–1 cells were cultured as described previously [18, 24]. These cells were mycoplasma-free and examined by short tandem repeat profiling at Mission Biotech (Taipei, Taiwan). BMS-345541 (50 mM prepared in dimethyl sulfoxide (DMSO) and stored at -80°C), an IKK allosteric site inhibitor [25], N-acetyl-L-cysteine (NAC, 10 mM freshly prepared in phosphate buffered saline (PBS) and stored at 4°C), a ROS scavenger, 3-methyladenine (3-MA, 100 mM in DMSO and stored at -80°C), a cell-permeable autophagic sequestration blocker [26], and chloroquine (CQ, 200 mM in water and stored at -80°C), blockage of autophagosome-lysosome fusion [27], were purchased from Sigma Aldrich (St. Louis, MO, USA).

### Irradiation of cell culture

Ionizing radiation (IR) was administered to cells by means of a RS2000 X-ray irradiator (Rad Source Technologies, Suwanee, GA, USA) at a dose rate of 86.24 mGy/sec. The medium was routinely changed after irradiation.

### Plasmids

The plasmids used for RNAi-mediated knockdown and ectopic expression of IGFBP3 were stably expressed as described previously [18].

### Immunoblot assay

Immunoblot assays were conducted as described previously [28]. The following primary antibodies were used: anti-IGFBP3 (1:1000, GTX113364, GeneTex, Irvine, CA, USA), anti-cytochrome c (1:1000, 556,433, BD Biosciences, Franklin Lakes, NJ, USA), anti-cleaved caspase 3 (1:500, IMG-144A, IMGENEX, San Diego, CA, USA), anti-light chain 3B (1:500, LC3B, GTX127375, GeneTex), anti-NF-κB (1:1000, #6956, Cell Signaling), anti-phosphorylated NF-κB (1:1000, #3033, Cell Signaling), anti-IκBα (1:1000, ab76429, Abcam, Cambridge, UK), anti-phosphorylated IκBα S32 (1:1000, ab92700, Abcam), and anti-α-tubulin (1:5000, GTX628802, GeneTex). Protein levels were determined by measuring the intensity of bands on western blots using ImageJ (National Institutes of Health, Bethesda, MD, USA). Protein levels were normalized against α-tubulin as an internal control. The relative ratio was calculated by dividing the normalized protein levels in stably expressing cells with that in control cells.

### Cell survival assay

The survival curves were created by calculating the mean value of absorbance at 490 nm using CellTiter 96(^R^) AQueous One Solution Cell Proliferation Assay (Promega, Madison, WI, USA) and a 96-well plate reader (Bio-Rad Laboratories, Hercules, CA), as described previously [29]. The relative cell viability was defined as the population of viable cells relative to controls, with the cell viability of control cells set to 1.

### Clonogenic assay

Cells (ranging from 50 to 10^4^ cells) were seeded into 6-well plates in duplicate for each dose. After irradiation, cells were incubated at 37 °C and 5% CO_2_ for 14 days. Cells were then fixed with methanol for 10 mins at room temperature and stained with 0.05% crystal violet for 30 mins. Only colonies containing more than 50 cells were counted. The relative surviving fraction (SF) is calculated as quotient of plating efficiency (treated) to PE (control): PE = number of colonies ÷ number of seeded cells; SF=PE (irradiated cells) ÷ PE (control cells).

### Orthotopic tumor model and in vivo irradiation

The procedure for orthotopic injection was described previously [18]. Briefly, 5 ~ 6-week-old male nude mice (BALB/cAnN.Cg-Foxn1^nu^/CrlNarl) were purchased from the National Laboratory Animal Center (Taipei, Taiwan) and anesthetized via inhalation of 5% Isoflurane (Piramal Critical Care, Bethlehem, PA, USA). Mice were randomized assigned to the groups. 5 × 10^5^ cells in 50 μl of sterile PBS were injected into the buccal mucosa of mice (*n* = 5–8). The tumor size was decided by measuring the tumor volume (mm^3^) with the formula 1/2 × (length) × (width)^2^ using the calipers. When the average tumor size reached 3–5 mm in diameter, the mice were locally irradiated with 8 Gy IR using an X-ray irradiator (RS2000, Rad Source Technologies) at dose rate of 22.98 mGy/sec. The animals were then sacrificed at the indicated time points, and the tumors were weighed, processed for formalin-fixed, paraffin-embedded (FFPE) tissues, stained with hematoxylin and eosin (H&E, Sigma Aldrich), and examined via histopathology.

### Immunohistochemistry

Immunohistochemistry (IHC) was performed with VECTASTAIN Elite ABC HRP kit (Vector lab, Burlingame, CA, USA) as previously described [30]. The following primary antibodies were used: anti-Ki-67 (1:500, NCL-Ki-67p, Novacastra Laboratories, Newcastle upon Tyne, UK), anti-NF-κB (1:500, sc-8008, Santa Cruz, Dallas, TX, USA) and anti-IL-6 (1:100, GTX110527, GeneTex). Chromogenic detection was performed with Vector NovaRED Substrate kit (SK-4800, Vector Lab). Sections were counterstained with hematoxylin and viewed under bright-field microscopy (Leica Microsystems, Wetzlar, Germany). For Ki-67 staining, 4–5 fields per specimen were observed at 200× magnification. Using ImageJ plugin ImmunoRatio imaging software, the percentage of positive Ki-67 staining was defined as the total intensity of positive nuclei of tumor cells divided by that of the total nuclei in the field. For analysis of nuclear NF-κB, total fields with nuclear signal were observed in each specimen at 400× magnification. The data were expressed as mean number of nuclear NF-κB per microscopic field. For assessment of NF-κB and IL-6 expression, quantitative scoring and analysis was used. The expression intensity was scored as follows: 0, negative; 1, faint to weak; 2, moderate; and 3, strong. The extent of expression was scored as percent of immunoreactivity-positive tumor cells as follows: 0, < 1%; 1, 1–10%; 2, 11–30%; 3, 31–50%; and 4, 51–100%. The final immunoreactivity score (IRS, 0 to 12) was the product of the intensity and the extent scores.

### Terminal deoxynucleotidyl transferase dUTP nick end labeling (TUNEL) assay

The in situ apoptosis detection kit (ab206386, Abcam) was used, according to the manufacturer’s instructions, to assess the level of apoptosis in paraffin-embedded tissue sections. Fifteen fields with apoptotic cells were observed in each specimen at 200× magnification. The data were expressed as mean number of apoptotic cells per microscopic field.

### Cell cycle analysis

Cells were trypsinized and fixed with 70% ethanol, followed by staining with propidium iodide (PI, Sigma Aldrich). The cellular DNA content of each sample was determined by flow cytometry (FACSCalibur, BD Biosciences, San Jose, CA, USA). All experiments were performed in duplicate.

### Apoptosis assay

A total of 2 × 10^5^ cells/well in 6-well plates were exposed to IR and then harvested at the indicated time points. The apoptosis assay was performed using the FITC Annexin V Apoptosis Detection Kit (BD Biosciences), according to the manufacturer’s instructions.

### γ-H2A histone family member X (γ-H2AX) staining

Cells were fixed with 4% paraformaldehyde (Sigma Aldrich) at 37°C for 30 mins, permeabilized with 0.1% Triton X-100 (Sigma Aldrich) and stained using the primary mouse monoclonal anti-γ-H2AX antibody (1:100, #05–636-1, Millipore) and the secondary anti-mouse Dylight™ 488- or 549-conjugated IgG (1:2000, 610–141-002/610–142-002, Rockland Immunochemicals, Limerick, PA, USA). Nuclei were stained with 4′,6-diamidino-2-phenylindole (DAPI, Invitrogen). The histone foci were visually counted using fluorescence microscopy at 200× or 400× magnification. The intensity of green or red pixels was calculated by the ImageJ software. The mean of intensity was adjusted by the cell number in the field.

### Cellular oxidative stress

The intracellular ROS level was assessed through measurement of 2′, 7′-dichlorodihydrofluorescein diacetate (DCF-DA) oxidation (Molecular Probes Inc., Waltham, MA, USA), which produces fluorescent 2′, 7′-dichlorofluorescein (DCF). Cells were incubated with culture medium containing 10 μM DCF-DA for 30 min at 37 °C, and then analyzed using a flow cytometer for DCF fluorescence intensity. A sample without DCF-DA was prepared as a negative control. The fluorescence values of test samples were subtracted by that of the negative control.

### Mitochondrial ROS

Cells were treated with 5 μM MitoSox Red (Invitrogen) in culture media for 30 mins at 37 °C, and then trypsinized and washed with warm PBS. The intensity of MitoSox Red fluorescence was determined by a flow cytometer.

### Mitochondrial membrane potential (MMP)

This assay was based on the detection of the MMP changes in cells by the cationic, lipophilic JC-10 dye. In normal cells, JC-10 concentrates in the mitochondrial matrix where it formed red fluorescent aggregates. However, in cells with MMP loss, JC-10 diffused out of mitochondria. It changed to monomeric form and stained cells in green fluorescence. Cells were incubated in culture medium containing 1 μM JC-10 (Sigma Aldrich), for 30 min at 37 °C. After resuspension in serum-free medium, the cells were analyzed using a flow cytometer. The JC-10 fluorescence intensity of monomeric form was normalized to that of aggregate form to calculate the ratio of monomeric/aggregate.

### Microarray and data analysis

Microarray analysis was performed as described previously [18], and the data are available in the Gene Expression Omnibus (GEO) under Accession No. GSE139023. Genes with raw signal < 100 were filtered out, and the remaining genes were analyzed further. Differentially expressed genes were selected based on expression of > 2-fold change. The canonical pathway analysis tool in Ingenuity Pathways Analysis (IPA, Ingenuity Systems, Redwood City, CA, USA) was used to identify signaling and metabolic pathways associated with differentially expressed genes. Gene set enrichment analysis (GSEA) was performed using the javaGSEA software developed at the Broad Institute (Cambridge, MA, USA) and the MSigDB Hallmark gene set collection. Gene sets with a nominal of *p*-value < 0.05 and false discovery rate (FDR) value ≤25% were considered to have significant enrichment.

### Quantitative reverse transcription polymerase chain reaction (qRT-PCR)

The qRT-PCR was performed as described previously [30]. The following primer sequences were used: IGFBP3-F: 5′ CAAGCGGGAGACAGAATATGG; IGFBP3-R: 5′ GGACTCAGCACATTGAGGAACTT; IL6-F: 5′ GGCAGAAAACAACCTGAACCTT; IL6-R: 5′ GGCAAGTCTCCTCATTGAATCC; IL1A-F: 5′ GGAGTCATTTCATTGGCGTTTGAGT, IL1A-R: 5′ GTCTTCAAACATGTCTGGAACTTTGGC; IL1B-F: 5′ CACCTTCTTTCCCTTCATCTTTG; IL1B-R: 5′ ATCCCATGTGTCGAAGAAGATAG; β-actin-F: 5′ TGGATCAGCAAGCAGGAGTATG; β-actin-R: 5′ GCATTTGCGGTGGACGAT. All amplifications were performed in triplicate.

### NF-κB reporter assay

The human embryonic kidney cell line HEK293T were plated at 2 × 10^5^ cells/well in 6-well plates and co-transfected with 0.5 μg of the firefly pNF-κB-luc luciferase vector (an NF-κB responsive gene), 0.1 μg of the *Renilla* luciferase gene pRL-TK (internal control) and IGFBP3 expressing plasmids. Cells were irradiated at 48 h post-transfection and lysates were collected in reporter lysis buffer using Dual luciferase reporter assay kit (E1910, Promega) at 4 h post-irradiation. Lysates were then subjected to freeze-thaw three times and centrifugation at 2000 rpm for 10 mins. Cell lysate supernatant samples were stored at − 70 °C until use in subsequent assays. Luciferase assay was measured according to the manufacturer’s protocol (Promega). The luciferase activity was calculated by normalizing firefly luciferase activity to that of *Renilla* luciferase.

### Bio-Plex cytokine assay

One hundred μl of conditioned medium, collected from 10^3^ cells/well in 96-well plate 24 h after exposure to 10 Gy IR, was prepared by centrifugation at 2000 rpm for 10 mins and used to analyze IL-1β, IL-6 and IL-8 levels via the microsphere-based Bio-Plex Suspension Array system (Bio-Rad Laboratories), according to the manufacturer’s instructions. The cytokine levels were adjusted relative to the numbers of cells collected, as determined by MTS assay using CellTiter 96(^R^) AQueous One Solution Cell Proliferation Assay.

### Statistical analysis

A Cox proportional hazards model was used to determine independent predictors of survival among factors that were deemed significant as covariates in the univariate analysis. The log rank test was used to evaluate the significance of differences in survival between the stratified groups. The Student’s t-test was used to assess the significance of differences between two groups in vitro experiments. For all comparisons, *p* < 0.05 was considered statistically significant.

## Results

### IGFBP3 enhances radiation-induced cell-killing in vitro

After assessing expression of IGFBP3 in 48 late-stage OSCC samples by IHC [18], we sought to explore the association between IGFBP3 expression and the clinical outcome of OSCC patients. Survival analysis using a Cox regression model, showed that IGFBP3 protein expression was strongly correlated with overall survival (HR: 0.3484; 95% confidence interval [CI] 0.1259–0.9637, *p* = 0.0422), indicating that OSCC patients with higher IGFBP3 expression had improved overall survival compared with those with lower IGFBP3 expression. Furthermore, when patients were stratified using IGFBP3 expression level as a threshold, Kaplan-Meier survival analysis showed that OSCC patients with low levels of IGFBP3 had a reduced survival rate compared to those with high IGFBP3 levels (*p* = 0.0336, Figure S1A). Our data suggest that IGFBP3 expression may play a role in response to conventional OSCC therapies, such as radiation and combination treatment with radiotherapy and chemotherapy.

**Fig. 1:**
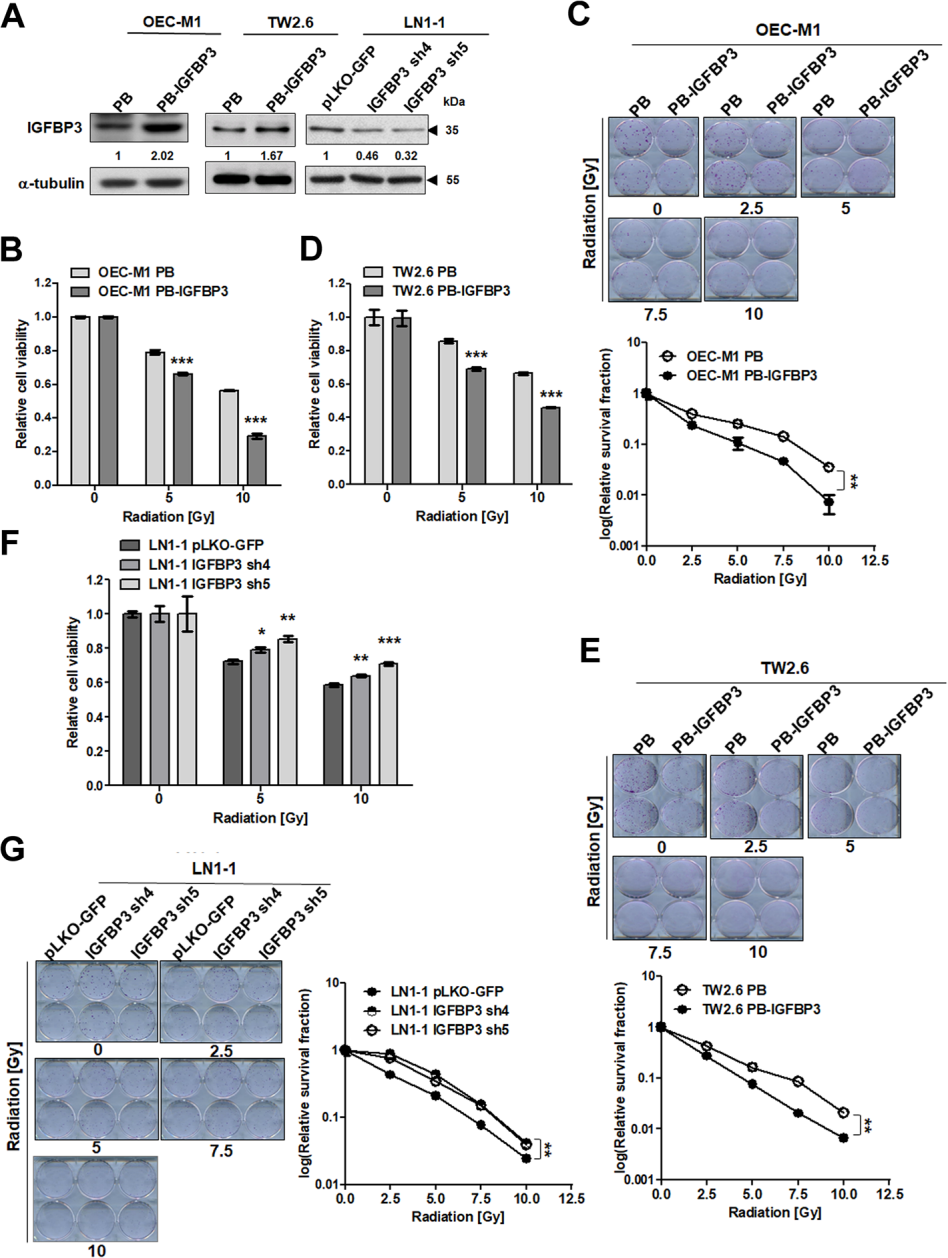
IGFBP3 enhanced IR-induced cell-killing in vitro. **a** Immunoblot analysis of IGFBP3 protein in OEC-M1 cells and TW2.6 cells with ectopic IGFBP3 expression (PB-IGFBP3) or vector control (PB) and in LN1–1 cells with IGFBP3 knockdown (IGFBP3 sh4 and sh5) and vector controls (pLKO-GFP). Protein levels were normalized to expression of α-tubulin, which served as an internal control. Relative ratios were determined by dividing the IGFBP3 protein level in each expression variant by that in the vector-expressing cells. **b** Effects of irradiation on survival and (**c**) colony formation of OEC-M1 and (**d** and **e**) TW2.6 cells with ectopic IGFBP3 expression versus vector controls and (**f** and **g**) LN1–1 cells with IGFBP3 knockdown and vector controls. Cells were treated with different doses of IR for viability and survival fraction using the MTS assay at 72 h and colony formation assay at 14 days after irradiation, respectively. The relative cell viability or survival fraction was normalized to corresponding untreated cells. Results from one of at least two independent experiments are shown. Values are expressed in mean ± SE. **p < 0.01; ***p < 0.001

To investigate the roles of IGFBP3 in response to OSCC treatment, we used OEC-M1 cells, which express low levels of IGFBP3, and generated OEC-M1 subclones with stable expression of IGFBP3 or the control vector. IGFBP3 expression in these subclones was confirmed by immunoblot assay (Fig. 1a). Following IR exposure (0–10 Gy), cell viability and colony formation were reduced in a dose-dependent manner at 72 h and 2 weeks after irradiation, respectively. Ectopic expression of IGFBP3 resulted in the reduced cell survival and colony formation after IR compared to corresponding controls (Fig. 1b, c). Following treatment with 0, 2.5, 5, 7.5, and 10 μM of the chemotherapeutic agent cisplatin for 72 h, the IGFBP3 expressing cells demonstrated resistance to cisplatin compared with control cells (Figure S1B). OEC-M1 cells with ectopic IGFBP3 expression demonstrated a reduced survival after cisplatin plus 10 Gy IR combination treatment compared with corresponding controls (Figure S1C). IGFBP3-mediated radiosensitivity was also consistently demonstrated in the TW2.6 OSCC cell line (Fig. 1d, e). In contrast to OEC-M1 cells, the IGFBP3-expressing TW2.6 cells showed increased sensitivity to cisplatin treatment compared to the control cells (Figure S1D). However, TW2.6 cells with ectopic IGFBP3 expression demonstrated worse survival after combination treatment with cisplatin and 10 Gy IR compared with corresponding controls (Figure S1E). These data indicate that high IGFBP3 expression enhances sensitivity to IR and may contribute to better outcomes after treatment of OSCC with conventional therapies. LN1–1 cells, which express high levels of IGFBP3, were stably transfected with different IGFBP3 shRNAs or the control vector (pLKO-GFP). The shRNA-mediated reduction of IGFBP3 expression was confirmed by western blot (Fig. 1a). LN1–1 cells with IGFBP3 knockdown showed improved survival and colony formation after IR treatment (Fig. 1f, g). Our data suggest that the IGFBP3 levels are positively associated with the radiosensitivity of OSCC cells.

### IGFBP3 enhances IR-induced cell-killing in vivo

**Fig. 2:**
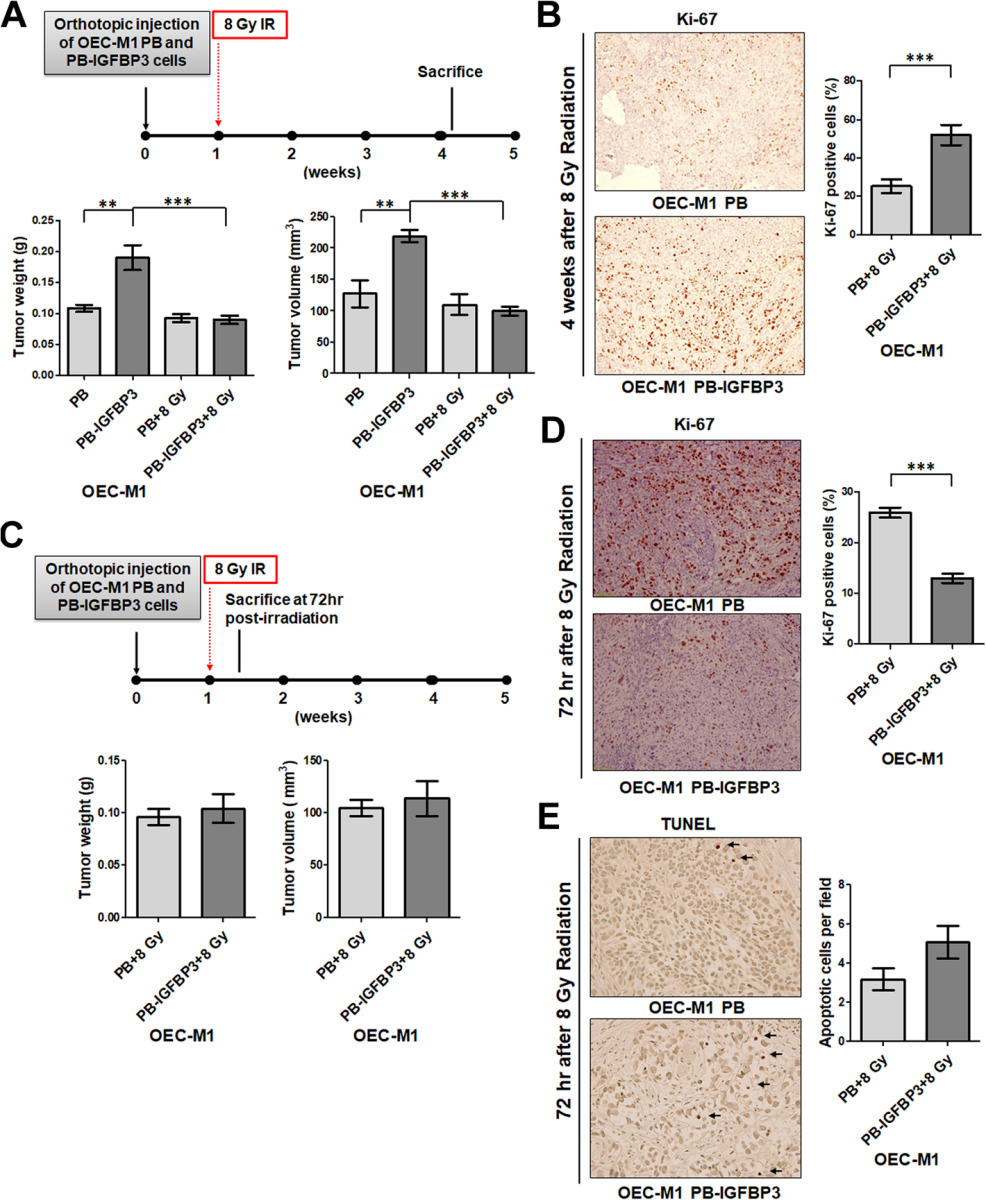
IGFBP3 enhanced IR-induced cell-killing in vivo. **a** Upper: The schematic for orthotopic xenograft with radiation (8 Gy IR) treatment and sacrifice 3–4 weeks after IR. Lower: Tumor weight (left panel) and volume (right panel) are shown for mice (*n* = 7–8) orthotopically injected with OEC-M1 cells with ectopic IGFBP3 expression (OEC-M1 PB-IGFBP3) or vector control (OEC-M1 PB) cells, with or without subsequent treatment with 8 Gy IR, and sacrificed 3–4 weeks after irradiation. **b** Left: Representative images of Ki-67-labeled OEC-M1 PB and PB-IGFBP3 tumor sections from mice treated with 8 Gy IR (200× magnification). Right: The mean percentage of Ki-67-positive cells per field. **c** Upper: The schematics for orthotopic xenograft with radiation (8 Gy IR) treatment and sacrifice 72 h after irradiation. Lower: Tumor weight (left panel) and volume (right panel) were shown for mice (*n* = 5) orthotopically injected with OEC-M1 PB or OEC-M1 PB-IGFBP3 cells, with subsequent treatment with 8 Gy IR, and sacrificed 72 h after irradiation. **d** Left: Representative images of Ki-67 and (**e**) TUNEL-labeled OEC-M1 PB and PB-IGFBP3 tumor sections from mice treated with 8 Gy IR and sacrificed 72 h after irradiation (200× magnification). Right: The mean percentage of Ki-67-positive and (**e**) TUNEL-positive cells per field. Black arrows indicate apoptotic cells. Results from one of at least two independent experiments are shown. Values are expressed in mean ± SE. **p < 0.01; ***p < 0.001

Based on the low survival fraction in clonogenic assay of 10 Gy-treated OSCC cells, orthotopic tumors at 3-5 mm in diameter were treated with or without 8 Gy IR (Fig. 2a, upper). After 4 weeks, the mean weight of non-irradiated tumors from mice receiving OEC-M1 cells with ectopic IGFBP3 expression (*n* = 8; 0.19 ± 0.02 g) was significantly greater than that of the irradiated tumors (*n* = 7; 0.09 ± 0.007 g, *p* = 0.0007; Fig. 2a, lower left). In contrast, the mean weight of vector-expressing OEC-M1 cell-derived orthotopic tumors from irradiated mice (*n* = 8; 0.109 ± 0.006 g), was similar to the mean weight of corresponding tumors from non-irradiated mice (*n* = 7; 0.093 ± 0.006 g; *p* = 0.0891; Fig. 2a, lower left). The volume of tumors from these mice followed the same pattern as tumor weight (Fig. 2a, lower right). Our results show that ectopic expression of IGFBP3 decreases tumor growth upon IR, suggesting a role for IGFBP3 in modulating tumor cell radiosensitivity in vivo. There was also an increase in Ki-67-positive cells in IGFBP3-expressing tumors (*n* = 16; 51.95 ± 5.239% per field) compared to controls (*n* = 16; 25.28 ± 3.479% per field; *p* = 0.0002; Fig. 2b).

At 72 h after IR, we observed similar weight and volume of orthotopic tumors in mice receiving OEC-M1 cells with ectopic IGFBP3 expression compared with vector controls (Fig. 2c). Immunostaining of tumor sections with an anti-Ki-67 antibody showed reduced Ki-67 staining in IGFBP3-expressing tumor sections (*n* = 25; 13 ± 0.9323% per field) compared to controls after IR (*n* = 25; 25.95 ± 0.9992% per field; *p* < 0.0001; Fig. 2d). Immunostaining of tumor sections by terminal deoxynucleotidyl transferase dUTP nick end labeling (TUNEL) assay demonstrated a higher trend of apoptotic cells in IGFBP3-expressing tumor cells (*n* = 75; 5.08 ± 0.8278% per filed) compared to controls (*n* = 75; 3.173 ± 0.56% per field; *p* = 0.0584; Fig. 2e) after IR. Our in vivo data indicate that ectopic IGFBP3 expression increases tumor cell apoptosis and inhibits tumor cell proliferation at 72 h after exposure to IR.

### IGFBP3 enhances IR-induced cell killing in vitro

**Fig. 3:**
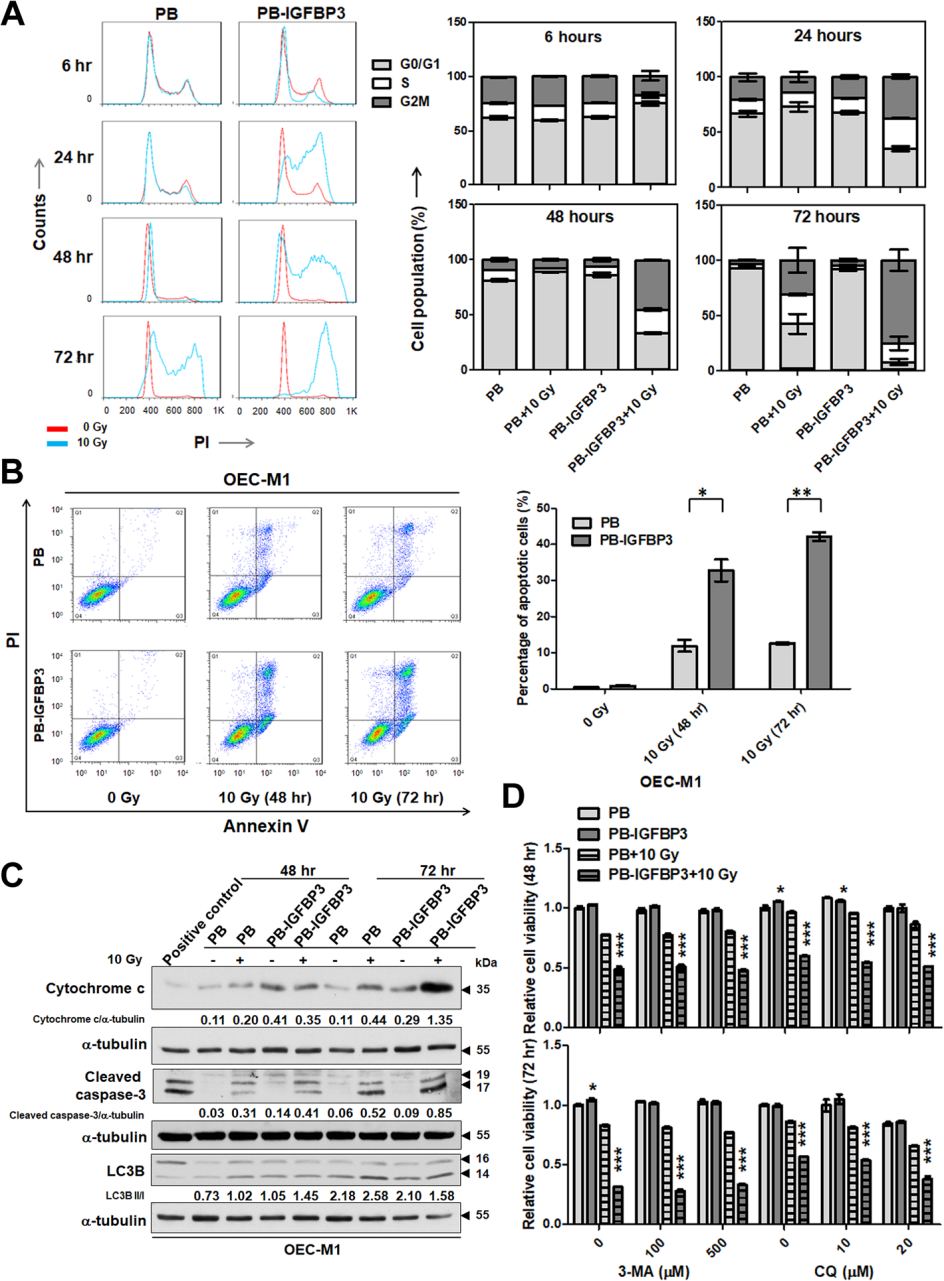
IGFBP3 enhanced IR-induced apoptosis. **a** Cell cycle analysis of OEC-M1 PB and OEC-M1 PB-IGFBP3 cells at 6, 24, 48 and 72 h after exposure to 10 Gy IR. Lfet: Representative DNA histograms of flow cytometric cell cycle analysis based on propidium idodide (PI) uptake. Red line: non-irradiated cells; blue line: irradiated cells. Right: Percentage of cells at each phase of the cell cycle. **b** Apoptosis assay of OEC-M1 PB and OEC-M1 PB-IGFBP3 cells treated with 10 Gy IR using Annexin V and PI. Upper: A representative diagram of flow cytometric analysis with different quadrants indicating different stages of apoptosis (lower left quadrant: healthy; lower right: early apoptosis; upper right: late apoptosis). Lower: Percentage of apoptotic cells following IR. **c** Immunoblot of cytochrome c, cleaved caspase-3, and LC3B in OEC-M1 PB and PB-IGFBP3 cells at 48 and 72 h after exposure to 10 Gy IR. The cells treated with 10 μM cisplatin served as a positive control. Protein levels were normalized to expression of α-tubulin, an internal control. The ratios of LC3B II/I were determined by dividing the LC3B II (14 KDa) level by LC3B I (16 kDa) from each cell. **d** Effects of different doses of 3-MA (100 and 500 μM) and CQ (10 and 20 μM) on survival of ectopic IGFBP3 or vector expressing cells with or without irradiation. Cell viability was decided using the MTS assay at 48 and 72 h after irradiation. The relative cell viability was normalized to corresponding untreated control cells. Results from one of at least two independent experiments are shown. Values are expressed in mean ± SE. *p < 0.05; **p < 0.01; ***p < 0.001

At 6, 24, 48 and 72 h after exposure to 10 Gy IR, ectopic IGFBP3 expression gradually reduced the proportion of cells in G0/G1 and increased G2/M phases of the cell cycle in comparison with control cells (Fig. 3a). An assay for apoptosis via double staining with Annexin V and PI showed that ectopic IGFBP3 expression resulted in a higher proportion of apoptotic cells compared with control cells at 48 and 72 h after exposure to 10 Gy IR (Fig. 3b). The proportion of PI−/Annexin V+ and PI+/Annexin V+ cells in OEC-M1 cells with ectopic IGFBP3 expression reached 42.16%, compared with 12.61% in OEC-M1 cells with vector control expression at 72 h (*p* = 0.00152; Fig. 3b), suggesting that ectopic IGFBP3 expression increased the radiosensitivity of OSCC cells via induction of apoptosis. The levels of cytochrome c and cleaved caspase-3, markers of apoptosis, were assessed by immunoblot and were increased in irradiated compared to non-irradiated cells at 48 and 72 h after 10 Gy IR (Fig. 3c). At 72 h post-irradiation, the ratios of cytochrome c/α-tubulin and cleaved caspase-3/α-tubulin in irradiated OEC-M1 cells with ectopic IGFBP3 expression were higher (1.35 and 0.85, respectively; Fig. 3c) than those in the irradiated control cells (0.44 and 0.52, respectively; Fig. 3c), indicating that ectopic IGFBP3 expression increases mitochondria-associated apoptosis upon IR exposure. At 72 h after irradiation, the ratio of LC3B II/I, a marker for autophagy, in irradiated IGFBP3 expressing OEC-M1 cells was not higher (1.58; Fig. 3c) to that in the irradiated control cells (2.58; Fig. 3c). Additionally, treatment of different concentrations of 3-methyladenine (3-MA), a cell-permeable autophagic sequestration blocker, as well as chloroquine (CQ), blockage of autophagosome-lysosome fusion, did not increase the survival of irradiated IGFBP3 expressing OEC-M1 cells 48 or 72 h after 10 Gy IR (Fig. 3d). Our data indicated that autophagy is not involved in IGFBP3-mediated radiosensitivity.

### Increased IR-induced apoptosis in IGFBP3-expressing cells involves the mitochondrial pathway of apoptosis and ROS production

**Fig. 4:**
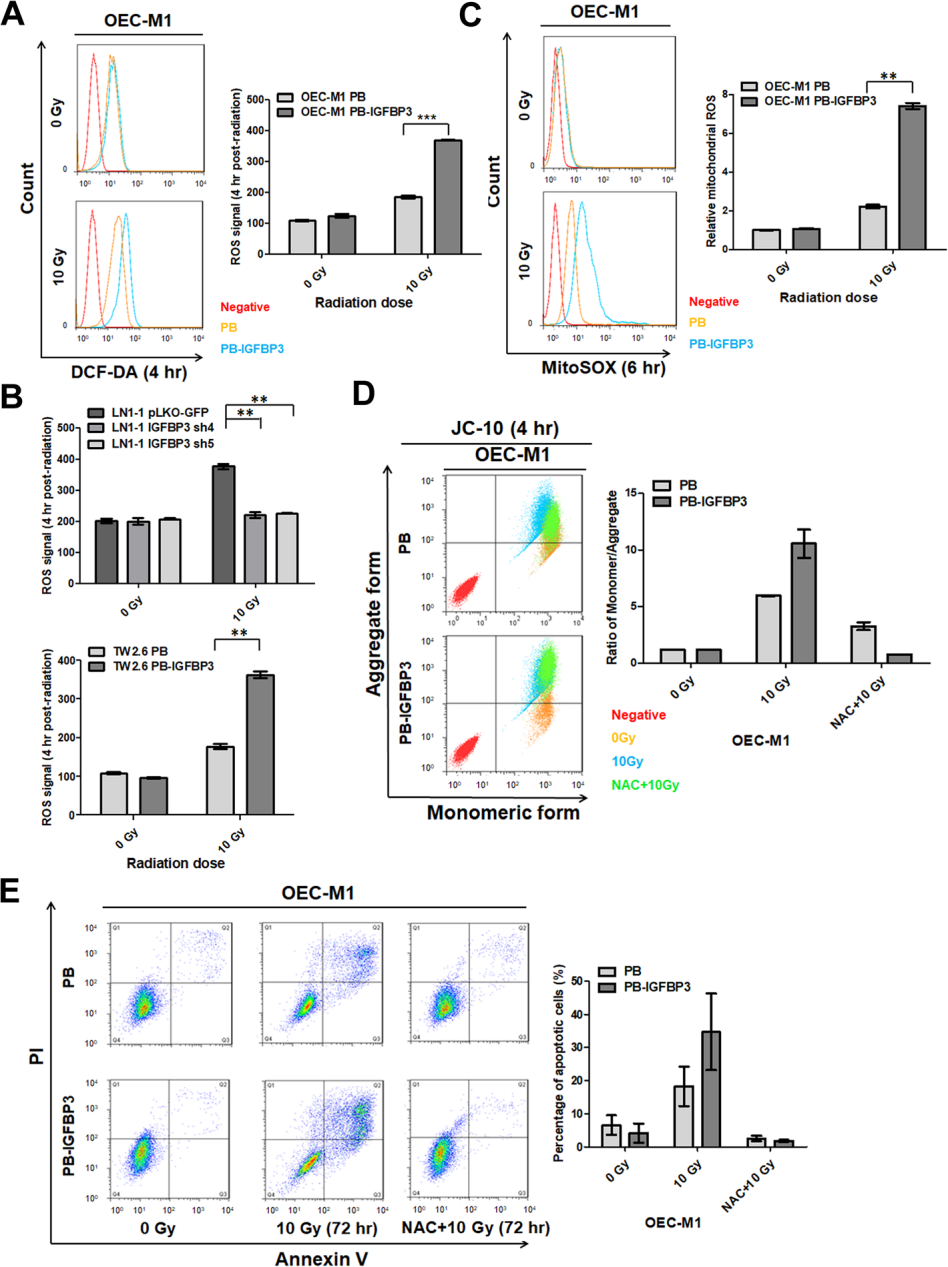
Increased IR-induced apoptosis in OSCC cells with ectopic IGFBP3 expression involves ROS production. **a** Analysis of ROS production at 4 h after irradiation of IGFBP3-expressing (PB-IGFBP3) or control (PB) cells with 10 Gy IR. Left: Representative histograms of ROS levels in different cell types (red line: negative control; orange line: vector expressing cells; blue line: IGFBP3 expressing cells). Right: The ROS signal was determined using H_2_DCF-DA. **b** Upper: Analysis of ROS production in LN1–1 cells with IGFBP3 knockdown (IGFBP3 sh4 and sh5) and control cells (pLKO-GFP); Lower: ROS production in TW2.6 cells with ectopic IGFBP3 expression (PB-IGFBP3) and control cells (PB). **c** Analysis of mitochondrial ROS production at 6 h after exposure to 10 Gy IR. Left: Representative histograms of mitochondrial ROS levels in different cell types (as described in part A above). Right: Relative mitochondrial ROS signal was calculated by dividing the normalized levels in IGFBP3-expressing cells or irradiated control cells by that in non-irradiated control cells. **d** Analysis of mitochondrial membrane potential (MMP) alteration in OEC-M1 PB and PB-IGFBP3 cells at 4 h after exposure to 10 Gy IR and NAC treatment. Left: Representative scatter plots of MMP levels in different cell types and treatment combinations (red: negative control; orange: non-irradiated cells; blue: irradiated cells; green: irradiated NAC-treated cells). Right: The relative ratio of monomeric/aggregate forms of JC-10 was obtained by dividing the normalized ratios in irradiated IGFBP3-expressing or control cells, with or without NAC treatment, by that in non-irradiated control cells. **e** Analysis of apoptosis in cells treated with 10 Gy IR, with or without NAC. Left: A representative diagram of flow cytometric analysis with different quadrants indicating different stages of apoptosis (lower left quadrant: healthy; lower right: early apoptosis; upper right: late apoptosis). Right: Percentage of apoptotic cells after exposure to IR with or without co-treatment with NAC. Results from one of at least two independent experiments are shown. Values are expressed in mean ± SE. **p < 0.01; ***p < 0.001

We used the γ-H2AX assay to investigate whether ectopic IGFBP3 expression affects IR-induced apoptosis by directly inducing DNA damage. We found equal γ-H2AX signal per cell when comparing IGFBP3- and vector-expressing cells at 72 h after exposure to 10 Gy IR (Figure S2A). Interestingly, the higher γ-H2AX signal per cell was detected in IGFBP3 expressing cells when compared to that in vector control cells at 0.5, 1, and 2 h after IR exposure (Figure S2B), indicating that DNA damage is occurred in IGFBP3-mediated radiosensitivity after IR exposure and repaired later. We also determined whether ectopic IGFBP3 expression promotes radiosensitivity through regulation of cellular ROS levels [31]. A higher level of ROS production was detected in OEC-M1 cells with ectopic IGFBP3 expression compared with control cells at 2, 4, and 6 h after exposure to 10 Gy IR (Figures S3A, 4a, S3B). The difference in ROS generation in IGFBP3-expressing cells was nearly double that of control cells at 4 h after irradiation (Fig. 4a). IR-induced ROS production in LN1–1 cells was suppressed by IGFBP3 knockdown (Fig. 4b), while ROS generation in TW2.6 cells was enhanced by ectopic IGFBP3 expression (Fig. 4b). These data suggest that IGFBP3 expression regulates IR-induced ROS production.

Mitochondria are vulnerable to various ROS stimuli, and we found more mitochondrial ROS production in OEC-M1 cells with ectopic IGFBP3 expression than in control cells at 4 h and 6 h after exposure to 10 Gy IR (Figures S3C, 4c). At 6 h after irradiation, ectopic IGFBP3 expression increased the mitochondrial ROS level by 3.5 folds in OEC-M1 cells compared to controls (*p* = 0.0012; Fig. 4c). In assessment of mitochondrial membrane potential (MMP), we found a significantly disruption of MMP in IGFBP3 expressing OEC-M1 and control cells after exposure to 10 Gy IR compared to corresponding non-irradiated cells (*p* = 0.067; Fig. 4d). Treatment with the antioxidant N-acetyl-L-cysteine (NAC) suppressed the IR-induced increase in MMP of both IGFBP3- and vector-expressing cells (Fig. 4d). Also, NAC treatment abolished IR-induced DNA damage in both IGFBP3 and vector-expressing cells at 0.5, 1 and 2 h after treatment of 10 Gy irradiation (Figure S2C). IR-induced apoptosis was also significantly suppressed by NAC administration in both IGFBP3- and vector-expressing cells at 72 h after exposure to 10 Gy IR (Fig. 4e). Similarly, we observed that the ROS scavenger abolished IR-induced apoptosis in IGFBP3 expressing TW2.6 cells (Figure S4A). The data indicated that the increased ROS production was required for IGFBP3 enhancement of IR-induced DNA damage, MMP increase, and cell apoptosis.

### IGFBP3 activates NF-κB signaling

**Fig. 5:**
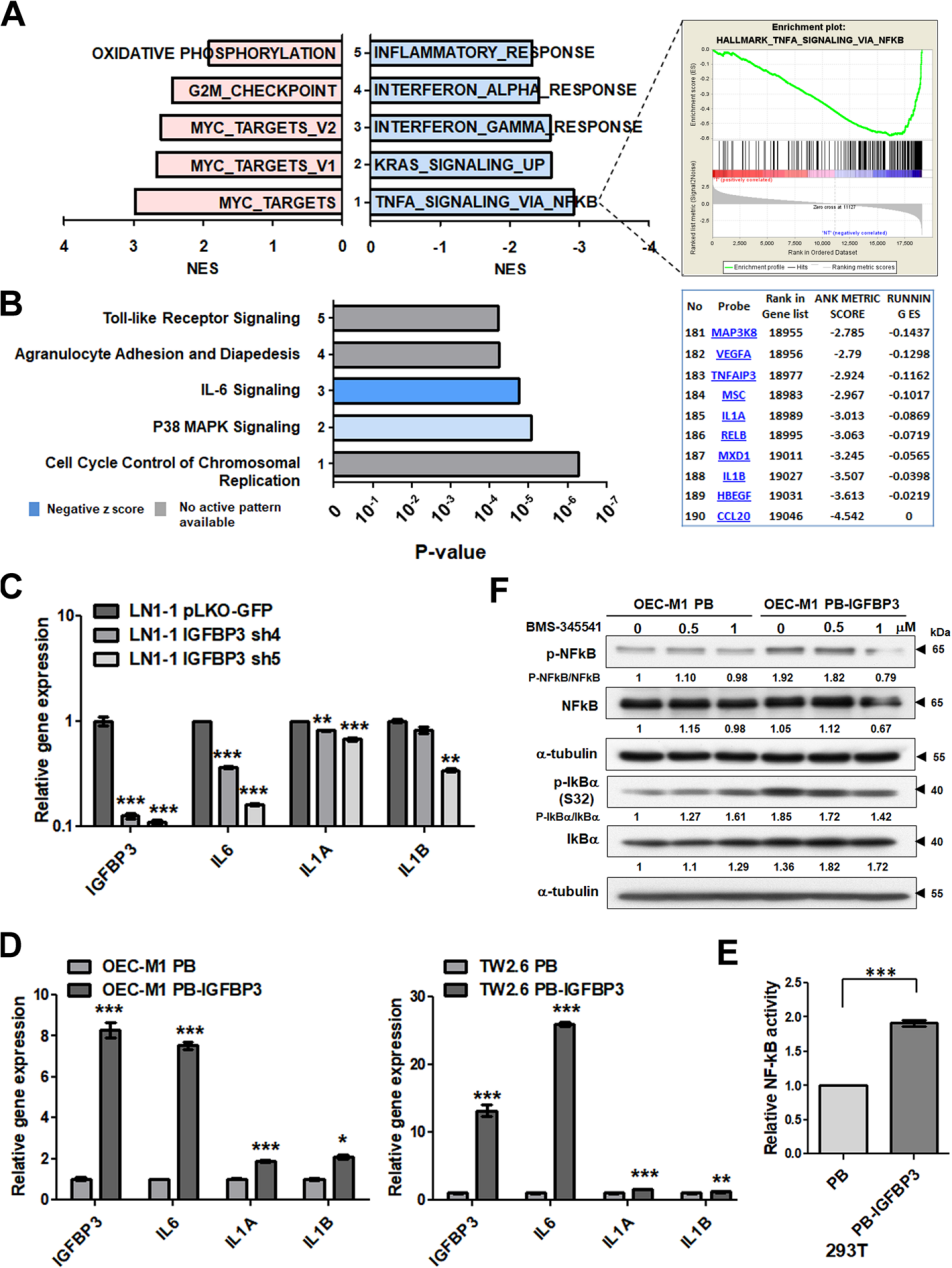
NF-κB signaling was activated by ectopic IGFBP3 expression. **a** Upper left: The most enriched pathways associated with IGFBP3 knockdown in LN1–1 cells by GSEA analysis. Light blue indicates a negative normalized enrichment score (NES) and pink indicates a positive NES. Upper right: GSEA analysis of IGFBP3 knockdown versus vector control LN1–1 cells with the HALLMARK TNFA signaling via NF-κB pathway. Lower right: List of more differentially expressed genes in the pathway. **b** The most statistically significant canonical pathways associated with IGFBP3 knockdown in LN1–1 cells by IPA. Blue indicates a negative z score; grey indicates no active pattern. **c** Relative levels of *IGFBP3*, *IL6*, *IL1A*, and *IL1B* mRNA in LN1–1 cells with IGFBP3 knockdown and (**d**) OEC-M1 and TW2.6 cells with ectopic IGFBP3 expression. All amplifications were normalized to β-actin. The relative mRNA expression in cells with IGFBP3 knockdown or ectopic IGFBP3 expression was normalized to that in corresponding control cells. **e** Relative NF-κB activity in 293T cells with ectopic IGFBP3 expression was assessed using the dual luciferase reporter assay, with the activity of 293T cells expressing the control vector set to 1. **f** Immunoblot assay of p-NF-κB, NF-κB, p-IkBα and IkBα in OEC-M1 PB and OEC-M1 PB-IGFBP3 cells with or without IKK inhibitor (BMS-345541) treatment. α-tubulin served as an internal control. Relative expression ratios were determined by dividing the normalized protein levels in BMS-345541-treated IGFBP3-expressing or control cells by that in untreated control cells. Results from one of at least two independent experiments are shown. Values are expressed in mean ± SE. *p < 0.05; **p < 0.01; *** *p* < 0.001

We performed microarray analysis to obtain differential gene expression profiles between IGFBP3 knockdown and control cells (Figure S5A). The dataset is available in GEO/GSE139023 dataset. Gene set enrichment analysis (GSEA) using the MSigDB Hallmark gene set collection indicated “TNFA_signaling_via_NF-κB” as the top negatively associated pathway enriched in differentially expressed genes (Fig. 5a). We also found that IGFBP3 expression was negatively correlated with NF-κB-related pathways in C2 curated data sets, including “Zhang_Response_to_IKK_inhibitor_and_TNF_Up” and “HINATA_NF-κB_Targets_Keratinocyte_Up” (Figure S5B). These data demonstrate down-regulation of NF-κB signaling in IGFBP3 knockdown cells. A total of 2436 probes showed a change of gene expression in IGFBP3 knockdown cells of at least 2-fold in comparison with the control cells, including 1386 up-regulated and 1050 down-regulated genes (Figure S5A). By Ingenuity Pathway Analysis (IPA), the canonical pathway analysis showed that “IL-6 signaling” was inhibited by IGFBP3 knockdown (z score = − 2.887; Fig. 5b). These data support prior reports suggesting that NF-κB activation is tightly regulated by IGFBP3 expression [32, 33].

To confirm the relationship between IGFBP3 expression and NF-κB signaling as indicated by GSEA and IPA, quantitative reverse transcription polymerase chain reaction (qRT-PCR) was used to evaluate the expression of *IL6*, *IL1A* and *IL1B* and demonstrated down-regulation of these genes by IGFBP3 knockdown in LN1–1 cells (Fig. 5c). Inversely, ectopic IGFBP3 expression in OEC-M1 and TW2.6 cells was associated with increased expression of *IL6*, *IL1A* and *IL1B* (Fig. 5d). Analysis via the Bio-Plex assay also demonstrated effects of IGFBP3 on NF-κB signaling by showing upregulated expression of IL-1β, IL-6 and IL-8 protein in ectopic IGFBP3 expressing OEC-M1 and TW2.6 cells, and down-regulation of these proteins in IGFBP3 knockdown LN1–1 cells (Figure S6). These data indicate that levels of cytokines, such as IL-1β, IL-6 and IL-8, are positively regulated by IGFBP3 expression.

Using the NF-κB luciferase reporter assay in 293T cells, we found that the IGFBP3 expressing plasmids increased NF-κB activity when compared to the control vector (Fig. 5e). The western blot analysis showed that ectopic IGFBP3 expression elevated the phosphorylation status of nuclear factor of IκBα and p65-NF-κB (Fig. 5f). In contrast, treatment of IκB kinase (IKK) inhibitor, BMS-345541 suppressed the phosphorylation of IκBα and p65-NF-κB in a dose-dependent manner in IGFBP3 expressing OEC-M1 cells (Fig. 5f). These data strongly suggested that ectopic IGFBP3 expression constitutively elevated canonical NF-κB activity in OEC-M1 cells.

### IGFBP3-mediated ROS production is stimulated by NF-κB activation and IL-6 expression

**Fig. 6:**
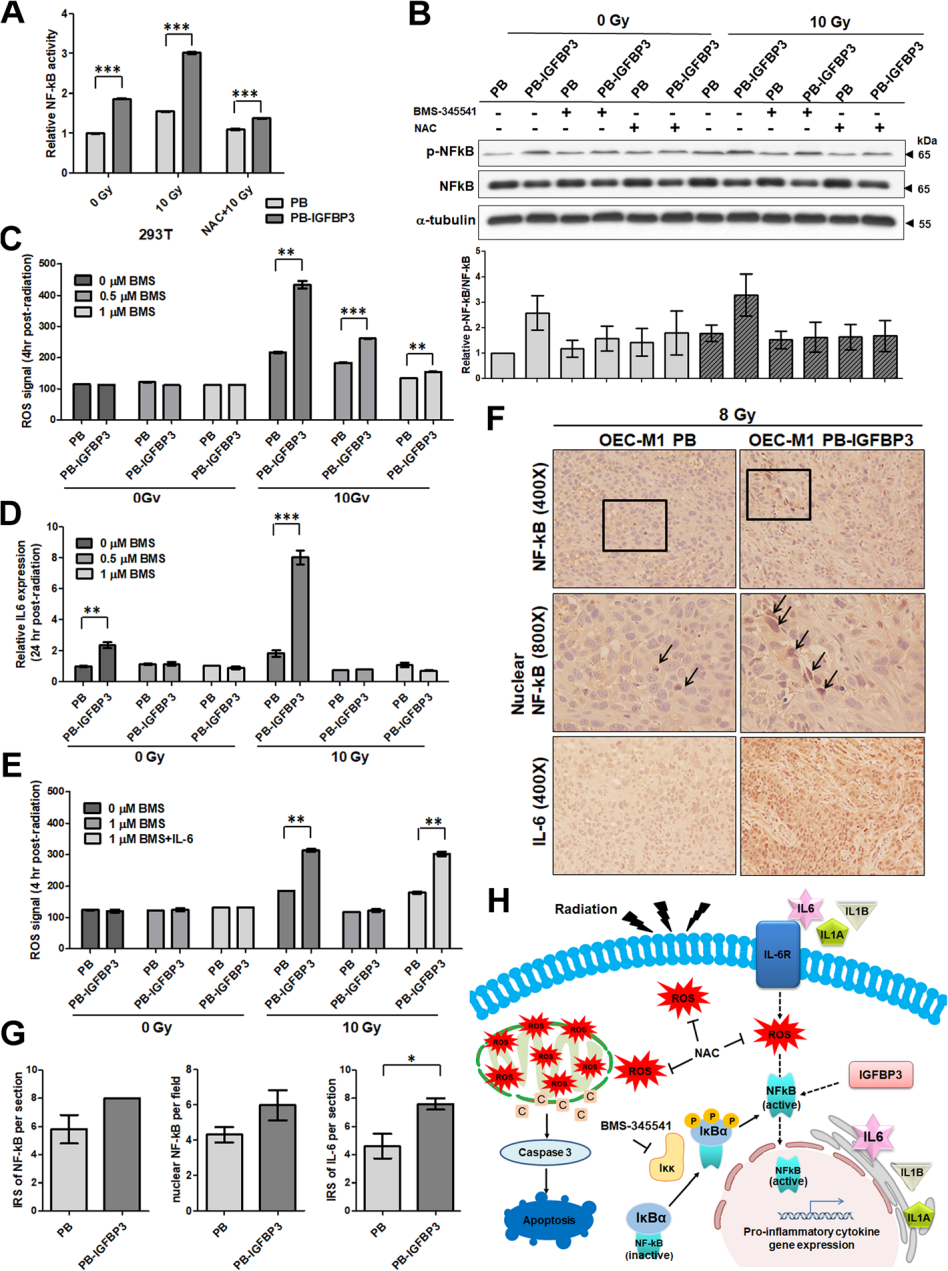
IGFBP3-mediated ROS production was stimulated by NF-κB activation and IL-6 expression. **a** NF-κB activity in 293T cells expressing either IGFBP3 or the control vector with or without NAC pre-treatment and exposure to 10 Gy IR. NF-κB activity was assessed via the dual luciferase reporter assay and the relative activity was calculated by dividing the activity level in cells from each experimental condition by that in non-irradiated vector control cells. **b** Upper: Immunoblot of p-NF-κB and NF-κB in OEC-M1 PB and OEC-M1 PB-IGFBP3 cells with or without IR exposure or treatment with the IKK inhibitor BMS-345541 or the ROS scavenger NAC. Lower: Relative expression ratios were calculated by dividing the normalized protein levels in cells from each experimental condition by that in non-irradiated, untreated vector control cells. Data reflect the mean expression levels from three independent experiments. **c** ROS production at 4 h after exposure to 10 Gy IR in untreated or BMS-345541 (BMS)-treated OEC-M1 cells expressing IGFBP3 (PB-IGFBP3) or the control vector (PB) as assessed by flow cytometry. **d** The *IL6* level at 24 h after exposure to 10 Gy IR in untreated or BMS-345541 (BMS)-treated OEC-M1 cells expressing IGFBP3 (PB-IGFBP3) or the control vector (PB). All amplifications were normalized to β-actin. The relative mRNA expression in cells with ectopic IGFBP3 expression was normalized to that in untreated control cells. **e** Recombinant IL-6 rescued BMS-345541-suppressed ROS production. ROS signal at 4 h after exposure to 10 Gy IR in BMS-345541 (BMS) or IL-6 treated OEC-M1 cells expressing IGFBP3 (PB-IGFBP3) or the control vector (PB) was detected by flow cytometry. **f** IHC examination of tissues from irradiated mice with orthotopic PB and PB-IGFBP3-expressing tumors. Tissue sections were stained using anti-NF-κB and anti-IL-6 antibodies (400× and 800× magnification). Black arrows indicate nuclear NF-κB staining. **g** Quantification of NF-κB (left), nuclear NF-κB (middle) and IL-6 (right) expression in PB and PB-IGFBP3-expressing tumors at 72 h after exposure to 8 Gy IR. Immunoreactivity scores (IRSs) for NF-κB and IL-6 in PB and PB-IGFBP3 tumor sections are shown, and the number of cells positive for nuclear NF-κB per field is indicated. **h** Illustration of the role of IGFBP3 in enhancement of radiosensitivity in OSCC cells via the NF-κB/IL-6/ROS signaling axis. Ectopic IGFBP3 expression enhances NF-κB activity, induces expression of inflammatory cytokines, such as IL-1β, IL-8 and IL-6. Upon IR, ROS initiates the axis of NF-κB/IL-6/ROS and forms a positive feedback. The ROS is highly amplified in IGFBP3-expresing cells upon IR and lead to cell apoptosis via mitochondria-dependent cell death. Results from one of at least two independent experiments are shown. Values are expressed in mean ± SE. *p < 0.05; **p < 0.01; ***p < 0.001

Both ectopic IGFBP3 expression and IR treatment elevated NF-κB activity in 293T ells (Fig. 6a). Similarly, the levels of IL-1β, IL-6 and IL-8 in OSCC cells were enhanced by ectopic IGFBP3 expression and IR exposure (Figure S6). These data indicate that both ectopic IGFBP3 expression and IR enhance NF-κB signaling and increase downstream inflammatory cytokine production. However, NAC treatment suppressed NF-κB activity (Fig. 6a). Similarly, the level of phosphorylated NF-κB was increased by ectopic IGFBP3 expression and irradiation, while NAC administration suppressed the phosphorylation of p65-NF-κB (Fig. 6b). Our data suggest that ectopic IGFBP3 expression and ROS are the key players in regulation of NF-κB activity.

ROS production in IGFBP3 expressing OEC-M1 cells upon IR was inhibited by BMS-345541 in a dose-dependent manner (Fig. 6c). The levels of *IL6* expression in irradiated IGFBP3 expressing OEC-M1 and TW2.6 cells were abolished by BMS-345541 (Figs. 6d, S4B). The data indicated that NF-κB signaling is required for IGFBP3-mediated ROS production and up-regulation of *IL6*. Treatment with exogenous IL-6 had no impact on ROS production in BMS-345541-treated OSCC cells without irradiation, however, IL-6 restored ROS production in BMS-345541-treated IGFBP3-expressing OEC-M1 cells upon IR (Fig. 6e). Comparable results were shown in TW2.6 cells (Figure S4C). These data demonstrate that ectopic IGFBP3 expression enhances ROS production via activation of NF-κB signaling and downstream cytokine expression.

Immunostaining of orthotopic tumor sections with an anti-NF-κB antibody showed similar levels of NF-κB-positive staining in irradiated IGFBP3-expressing tumor cells (*n* = 5; Immunoreactivity score (IRS): 8 per section) compared to controls (*n* = 5; IRS: 5.8 ± 1.02 per section; Fig. 6f, g). However, the levels of nuclear NF-κB signal were higher in irradiated IGFBP3-expressing tumor cells (*n* = 60; 5.967 ± 0.857 per field) than in those in control sections (*n* = 60; 4.3 ± 0.4287 per field; *p* = 0.0846; Fig. 6f, g). Additionally, immunostaining for IL-6 demonstrated significant upregulation of IL-6 in irradiated IGFBP3-expressing tumor cells (*n* = 5; IRS: 4.6 ± 0.8718 per section) relative to controls (*n* = 5; IRS: 7.6 ± 0.4 per section; *p* = 0.0143; Fig. 6f, g). Our in vivo data indicated that ectopic IGFBP3 expression increased the levels of nuclear NF-κB and IL-6 upon IR.

## Discussion

Radiotherapy is a standard adjuvant treatment for locally advanced OSCC, therefore therapeutic strategies that increase the radiosensitivity of OSCC without increasing toxicity are needed. We found that OSCC patients with high IGFBP3 levels had better prognostic outcomes compared with patients with low levels of IGFBP3 (Figure S1A). The stage I non-small cell lung cancer patients, whose tumors harbor the high IGFBP3 expression caused by an unmethylated promoter, had a trend towards increased disease-free survival [34, 35]. Likewise, Lou et al. reported that a high IGFBP3 level was associated with favorable radiotherapy response and good prognosis among patients with esophageal squamous cell carcinoma [36]. These data encouraged us to investigate the relationship among IGFBP3 protein levels, radiotherapy, and chemotherapy. Our results indicate that ectopic IGFBP3 expression leads to decreased OEC-M1 and TW2.6 cell survival when treated with IR alone or in combination with cisplatin (Figs. 1b, c, d, e, S1C, S1E). Although loss of IGFBP3 expression results in the reduction of tumor cell sensitivity to cisplatin in non-small cell lung cancers [34], we did not consistently observe similar effects of IGFBP3 on cisplatin resistance in OEC-M1 and TW2.6 cells (Figures S1B, D). Our results showed that the levels of IGFBP3 affect the radiosensitivity of OSCC cells (Fig. 1f, g). Previously, IGFBP3 was proposed as a key radiosensitizer for improvement of individualized OSCC management [20]. Differently, Sakata et al. demonstrated that enhanced IGFBP3 expression decreased radiosensitivity of OSCC cells [21]. These data suggested that the dual roles of IGFBP3 in radiosensitivity and radioresistance might be caused by different etiology of cancers.

We observed that the higher level of Ki-67 in IGFBP3-expressing tumors than that in control tumors 4 weeks after IR, indicating that the higher proliferation index of IGFBP3-expressing tumors might possibly lead to tumor regrowth at a later time point (Fig. 2b). In Fig. 2d, the lower proliferation index of IGFBP3-expressing tumors at 72 h after IR indicates a radiation-induced proliferation stop, normally seen at this time point and leading to an enhanced induction of apoptosis. The remaining or radioresistant tumor cells showed a higher proliferation index 4 weeks after IR, perhaps as a mechanism to compensate the tumor mass deprivation (Fig. 2b).

Wu et al. demonstrated that IGFBP3 induced apoptosis through induction of cell cycle arrest at the G1/S phase in breast cancer cells [37]. In contrast, our data demonstrated a G2/M accumulation caused by ectopic IGFBP3 expression upon irradiation in p53-mutated OEC-M1 cells (Fig. 3a) [38]. Ectopic IGFBP3 expression significantly increased the percentage of apoptosis detected by Annexin V/PI staining in irradiated cells in vitro (Fig. 3b), and similar results were apparent in vivo via tissue analysis using the TUNEL assay (Fig. 2e). Although Ho et al. reported that cordycepin (3′-deoxyadenosine), a major bioactive component in Cordyceps sinensis, enhanced radiosensitivity in OSCC cells by inducing apoptosis and autophagy [39], our data showed that IGFBP3 enhancement of IR-induced apoptosis was dependent on the mitochondria pathway but independent of autophagy (Fig. 3c, d). Since IGFBP3 shuttles from the nucleus to the mitochondrial membranes and endoplasmic reticulum [40], we cannot rule out the possibility that IGFBP3 directly regulates apoptotic effects via mitochondrial shuttles.

Several studies reported that IGFBP3 may have a significant role in regulating DNA repair by forming a complex with the catalytic subunit of DNA-dependent protein kinase (DNA-PKcs) and epidermal growth factor receptor (EGFR) in response to DNA damage in breast cancer cells and OSCC cells [21, 41], implicating a direct role of IGFBP3 in DNA repair. However, we found that ectopic IGFBP3 expression enhanced IR-induced γ-H2AX via ROS production, indicating IGFBP3 served as an indirect player in IR-mediated DNA damage (Figure S2C). There is an enhanced foci number independent from IGFBP3 expression 72 h after IR (Figure S2A), suggesting that the remaining cells which are non-apoptotic and vital, might have an IR-enhanced proliferation and increased γ-H2AX signal. Additionally, our data indicate that IGFBP3 enhances ROS production after IR exposure and induces cell apoptosis via mitochondrial destruction (Fig. 4). In contrast, Natsuizaka et al. demonstrated that IGFBP3 may have a novel IGF-independent antioxidant activity that suppresses ROS-mediated cytotoxicity in esophageal cancer [16]. These data suggest a connection between IGFBP3 and ROS.

Recent studies have provided evidence for potential crosstalk between IGFBP3 and the NF-κB signaling cascade [32, 42]. IGFBP3 significantly enhanced TRAIL-induced cell death in colonic carcinoma-derived cell lines by inhibiting NF-κB activation in response to apoptotic induction [32], and Kim et al. showed that IGFBP3 enhanced etoposide-induced cell growth inhibition by blocking the NF-κB signaling pathway in gastric cancer cells [43]. In contrast, our data demonstrates that IGFBP3 induces apoptosis after IR exposure by activating NF-κB signaling (Fig. 6a, b). NF-κB signaling induces the expression of various pro-inflammatory genes and also participates in regulation of inflammation [22]. The *IL6* gene promoter has been shown to be activated through NF-κB binding [44]. In our study, expression of pro-inflammatory cytokines, such as IL-6 and IL-1β, was elevated by IGFBP3 (Figs. 5d, S6). Lee et al. showed that IGFBP3 degraded IκBα and p65-NF-κB proteins through IGFBP3 receptor (IGFBP3R)-mediated inhibition of tumor necrosis factor-α, thereby blocking activation of NF-κB signaling cascades [33]. However, the underlying mechanisms by which IGFBP3 activates NF-κB signaling have yet to be elucidated.

NF-κB activity may be positively or negatively regulated by ROS, which affect both cytoplasmic and nuclear NF-κB through regulation of IκBs degradation, NF-κB DNA binding, NF-κB transcriptional activity, and chromatin remodeling [45, 46]. Our data demonstrate that IGFBP3-mediated NF-κB activation could be inhibited by removal of ROS, suggesting ROS induces NF-κB activation (Figs. 6a, b). On the other hand, inhibition of IGFBP3-induced NF-κB activation using an IKK inhibitor decreased ROS production after exposure to IR. These data suggest positive feedback regulation between ROS and NF-κB activation. However, it is still unclear whether ROS-induced NF-κB activation is like to the IGFBP3-mediated NF-κB activation observed in this study.

Dong et al. found that IL-6 stimulated intracellular ROS levels in liver cancer cell lines [47]. Similarly, our results suggest that blockage of NF-κB activation diminishes ROS production, an effect that is reversible by treatment with exogenous IL-6 (Fig. 6d). The NF-κB/IL-6 signaling is generally a pro-inflammatory and pro-survival pathway [48], however, IL-6-induced apoptosis was observed in STAT3 deficient cells [49]. Like to our study, the additional IL-6 induced by IR not only was secreted for the outside-in signaling, but also amplified mitochondrial ROS production [50]. Some studied demonstrated that IGFBP3 enhanced the BAX signaling, associated with ROS-induced mitochondrial dysfunction, and contributed to cell death [51, 52]. Considering data from our study and other reports, we suggested more ROS accumulation in irradiated OSCC cells via a positive loop of NF-κB activation and ROS production. The ROS burst overcame the basal NF-κB/IL-6 survival effect and finally resulted in OSCC cell death. Further investigation is required to characterize the detailed mechanisms involved in this signaling. Consistent with our in vitro findings, we found a similar association between IGFBP3 expression, NF-κB activation and IL-6 production in irradiated OSCC tumors (Fig. 6f, g).

## Conclusions

We found that ectopic IGFBP3 expression enhanced IR-induced cell-killing in vivo and in vitro. More specifically, ectopic IGFBP3 expression increased IR-induced apoptosis as indicated by elevated ROS production. After IR exposure, IGFBP3-induced NF-κB activation was inhibited by ROS removal. IGFBP3-mediated ROS production was blocked by IKK inhibitor treatment, while exogenous IL-6 rescued the NF-κB-inhibited, IGFBP3-mediated ROS production. Our data suggest that IGFBP3 promotes OSCC cell death via positive feedback regulation of ROS production by inducing NF-κB activation and cytokine expression (Fig. 6h).

## Supplementary Information

**Additional file 1: Figure S1.** IGFBP3 enhanced radiation-induced cell-killing. (A) An overall survival correlation analysis was performed for 48 late-stage OSCC patient samples expressing different levels of IGFBP3. Patients were stratified into low (no or weak staining) and high (moderate or strong staining) groups based on IGFBP3 expression level; the log rank test was applied to detect significant differences in survival between groups. (B) Effects of cisplatin treatment on survival of OEC-M1 and (D) TW2.6 cells with ectopic IGFBP3 expression (PB-IGFBP3) versus vector controls (PB). Cells were treated with 0, 2.5, 5, 7.5, or 10 μM cisplatin and (C and E) combinations of 0, 2.5, 5, 7.5, or 10 μM cisplatin with 10 Gy IR and assessed for viability evaluated using the MTS assay at 72 hr after treatment. Results from one of at least two independent experiments are shown. Values are expressed in mean ± SE. *p<0.05; **p<0.01; ***p<0.001.

**Additional file 2: Figure S2.** Ectopic IGFBP3 expression increased γ-H2AX in the early post-irradiation. (A) The incidence of γ-H2AX foci at 72 hr after 10 Gy IR exposure is shown. Left: Representative images of γ-H2AX staining of IGFBP3- and vector-expressing OEC-M1 cells irradiated with 10 Gy IR at 400× magnification. Green, γ-H2AX; blue, DNA stained with DAPI. Right: The mean signal intensity of γ-H2AX foci per cell. (B) The incidence of γ-H2AX foci in IGFBP3- and vector-expressing OEC-M1 cells at 0.5, 1, 2, 4 and 6 hr after 10 Gy IR exposure is shown. Upper: Representative images of γ-H2AX staining of IGFBP3- and vector-expressing OEC-M1 cells after 10 Gy IR irradiation at 200× magnification. Red, γ-H2AX; blue, DNA stained with DAPI. Lower: The mean signal intensity of γ-H2AX foci per cell at different time points post-irradiation. (C) The incidence of γ-H2AX foci in IGFBP3- and vector-expressing OEC-M1 cells treated with or without NAC at 0.5, 1 and 2 hr after 10 Gy IR exposure is shown. Lower: Representative images of γ-H2AX staining of IGFBP3- and vector-expressing OEC-M1 cells treated with NAC and 10 Gy IR at 200× magnification. Red, γ-H2AX; blue, DNA stained with DAPI. Upper: The mean signal intensity of γ-H2AX foci per cell at different time points post-irradiation. Results from one of at least two independent experiments are shown. Values are expressed in mean ± SE. *p<0.05; **p<0.01.

**Additional file 3: Figure S3.** Ectopic IGFBP3 expression increased ROS production. (A) ROS production detected by DCF-DA at 2 hr and (B) 6 hr after 10 Gy IR in IGFBP3 expressing (PB-IGFBP3) or control (PB) OEC-M1 cells. Left: Representative diagrams of flow cytometric detection of ROS levels of each cell type (red line: negative control; orange line: vector control cells; blue line: IGFBP3 expressing cells). Right: Relative ROS signal from IGFBP3-expressing and vector control OEC-M1 cells with or without IR exposure. (C) Detection of mitochondrial ROS using MitoSOX at 4 hr post-irradiation with 10 Gy IR. Left: Representative diagrams of flow cytometric detection of ROS levels of each cell type (as described in part A above). Right: Relative mitochondrial ROS signal from IGFBP-expressing and vector control OEC-M1 cells with or without IR exposure. Mitochondrial ROS signal was determined by dividing the normalized levels in irradiated IGFBP3-expressing cells or vector control cells by that in non-irradiated control cells. Results from one of at least two independent experiments are shown. Values are expressed in mean ± SE. **p<0.01; ***p<0.001.

**Additional file 4: Figure S4.** IGFBP3-mediated radiosensitivity in TW2.6 cells via the NF-κB/IL-6/ROS signaling axis. (A) Apoptosis assay using annexin V and propidium iodide (PI) in IGFBP3- and vector-expressing TW2.6 cells with or without IR exposure and NAC treatment. Left: A representative diagram of flow cytometric analysis with different quadrants indicating different stages of apoptosis (lower left quadrant: healthy; lower right: early apoptosis; upper right: late apoptosis). Right: Percentage of apoptotic cells following no irradiation or induction by 10 Gy IR with or without NAC treatment. (B) The *IL6* level at 24 hr after exposure to 10 Gy IR in untreated or BMS-345541 (BMS)-treated TW2.6 cells expressing IGFBP3 (PB-IGFBP3) or the control vector (PB). All amplifications were normalized to β-actin. The relative mRNA expression in cells with ectopic IGFBP3 expression was normalized to that in untreated control cells. (C) Rescue of BMS-345541-suppressed ROS production by recombinant IL-6 in IGFBP3-expressing TW2.6 cells following irradiation. Upper: Representative diagrams of flow cytometric detection of ROS levels of each cell type with or without BMS-345541 or BMS plus IL-6 (red line, no cells; blue, non-irradiated vector expressing cells; orange, non-irradiated IGFBP3 expressing cells; green, irradiated vector control cells; dark green, irradiated IGFBP3 expressing cells). Lower: Relative ROS signal from IGFBP3-expressing (PB-IGFBP3) and vector control (PB) TW2.6 cells at 4 hr after irradiation with or without 10 Gy IR with or without BMS-345541 or BMS plus IL-6. Results from one of at least two independent experiments are shown. Values are expressed in mean ± SE. **p<0.01; ***p<0.001.

**Additional file 5: Figure S5.** IGFBP3 knockdown inhibited NF-κB-related pathways. (A) The schematic for comparison of IGFBP3 knockdown cells with vector control cells via analysis using GSEA and IPA. (B) Upper: The most enriched datasets associated with IGFBP3 knockdown in LN1-1 cells by GSEA using C2 curated data sets. Light blue represents a negative normalized enrichment score (NES). Lower left: Enrichment plots of gene signatures of “ZHANG Response To IKK Inhibitor and TNF Up”. Lower right: Enrichment plots of gene signatures of “HINATA NF-κB Targets Keratinocyte Up”.

**Additional file 6: Figure S6.** Levels of IL-1β, IL-6 and IL-8 in conditioned medium from IGFBP3 knockdown LN1-1, IGFBP3-expressing OEC-M1, and IGFBP3-expressing TW2.6 cells and their corresponding control cells at 24 hr after irradiation with 10 Gy IR as detected using the Bio-Plex assay.

## Data Availability

The data generated or analyzed and included in this article are available from the corresponding author upon reasonable request.

## Abbreviations

3-MA: 3-methyladenine
CQ: Chloroquine
FACS: Fluorescence activated cell sorter
GSEA: Gene set enrichment analysis
H&E: Hematoxylin and eosin
IGFBP3: Insulin-like growth factor binding protein 3
IHC: Immunohistochemistry
IκB: Nuclear factor of kappa light polypeptide gene enhancer in B-cells inhibitor
IKK: IκB kinase
IL-6: Interleukin 6
IPA: Ingenuity Pathway Analysis
IR: Ionizing radiation
MMP: Mitochondrial membrane potential
NAC: N-acetyl-L-cysteine
NF-κB: Nuclear factor kappa-light-chain-enhancer of activated B cells
OSCC: Oral squamous cell carcinoma
PI: Propidium iodide
qRT-PCR: Quantitative reverse transcription-polymerase chain reaction
ROS: Reactive oxygen species
TUNEL: Terminal deoxynucleotidyl transferase dUTP nick end labeling
