# Correction: Insulin-like growth factor binding protein 3 promotes radiosensitivity of oral squamous cell carcinoma cells via positive feedback on NF-κB/IL-6/ROS signaling

**DOI:** 10.1186/s13046-026-03794-4

**Published:** 2026-08-12

**Authors:** Yu-Lin Chen, Ssu-Han Wang, Jenn-Ren Hsiao, Fang-Yu Tsai, Shih Sheng Jiang, Alan Yueh-Luen Lee, Hui-Jen Tsai, Ya-Wen Chen

**Affiliations:** 1 National Institute of Cancer Research, National Health Research Institutes, 35, Keyan Road, Zhunan Town, Miaoli County, 35053 Taiwan; 2 Department of Otolaryngology, College of Medicine, National Cheng Kung University Hospital, National Cheng Kung University, Tainan, Taiwan; 3 National Institute of Cancer Research, National Health Research Institutes, Tainan, Taiwan; 4 Graduate Institute of Basic Medical Science, China Medical University, Taichung, Taiwan

**Correction: J Exp Clin Cancer Res 40, 95 (2021)**

**https://doi.org/10.1186/s13046-021-01898-7**

Following publication of the article [1], the authors identified an error in the representative image shown in the left panel of Fig. 1G. The image for the 7.5 Gy group was inadvertently misplaced during figure assembly.

The corrected figure is provided below.

**Incorrect Fig. 1G**

**Fig. 1:**
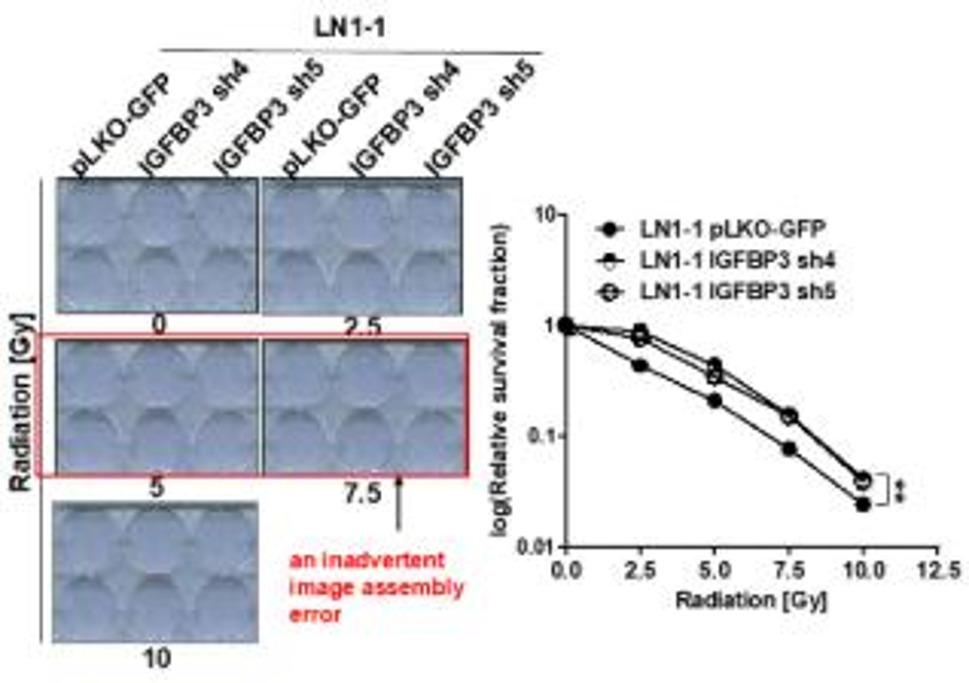
IGFBP3 enhanced IR-induced cell-killing in vitro. **a** Immunoblot analysis of IGFBP3 protein in OEC-M1 cells and TW2.6 cells with ectopic IGFBP3 expression (PB-IGFBP3) or vector control (PB) and in LN1–1 cells with IGFBP3 knockdown (IGFBP3 sh4 and sh5) and vector controls (pLKOGFP). Protein levels were normalized to expression of α-tubulin, which served as an internal control. Relative ratios were determined by dividing the IGFBP3 protein level in each expression variant by that in the vector-expressing cells. **b** Effects of irradiation on survival and (**c**) colony formation of OEC-M1 and (d and e) TW2.6 cells with ectopic IGFBP3 expression versus vector controls and (f and g) LN1–1 cells with IGFBP3 knockdown and vector controls. Cells were treated with different doses of IR for viability and survival fraction using the MTS assay at 72 h and colony formation assay at 14 days after irradiation, respectively. The relative cell viability or survival fraction was normalized to corresponding untreated cells. Results from one of at least two independent experiments are shown. Values are expressed in mean ± SE. **p < 0.01; ***p < 0.001

**Correct Fig. 1G**

**Fig. 1:**
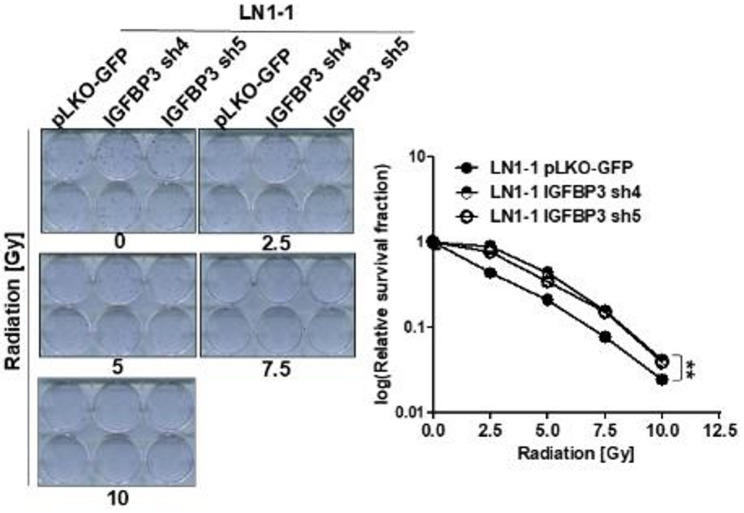
IGFBP3 enhanced IR-induced cell-killing in vitro. **a** Immunoblot analysis of IGFBP3 protein in OEC-M1 cells and TW2.6 cells with ectopic IGFBP3 expression (PB-IGFBP3) or vector control (PB) and in LN1–1 cells with IGFBP3 knockdown (IGFBP3 sh4 and sh5) and vector controls (pLKOGFP). Protein levels were normalized to expression of α-tubulin, which served as an internal control. Relative ratios were determined by dividing the IGFBP3 protein level in each expression variant by that in the vector-expressing cells. **b** Effects of irradiation on survival and (**c**) colony formation of OEC-M1 and (d and e) TW2.6 cells with ectopic IGFBP3 expression versus vector controls and (f and g) LN1–1 cells with IGFBP3 knockdown and vector controls. Cells were treated with different doses of IR for viability and survival fraction using the MTS assay at 72 h and colony formation assay at 14 days after irradiation, respectively. The relative cell viability or survival fraction was normalized to corresponding untreated cells. Results from one of at least two independent experiments are shown. Values are expressed in mean ± SE. **p < 0.01; ***p < 0.001

This correction does not affect the results, interpretation, or conclusions of the article. The original article [1] has been corrected.
