# Supplementary figures and images for: Insulin-like growth factor binding protein 3 promotes radiosensitivity of oral squamous cell carcinoma cells via positive feedback on NF-κB/IL-6/ROS signaling
Source: J Exp Clin Cancer Res. 2021 Mar 13;40:95. doi: 10.1186/s13046-021-01898-7 (PMC7955639; doi:10.1186/s13046-021-01898-7)

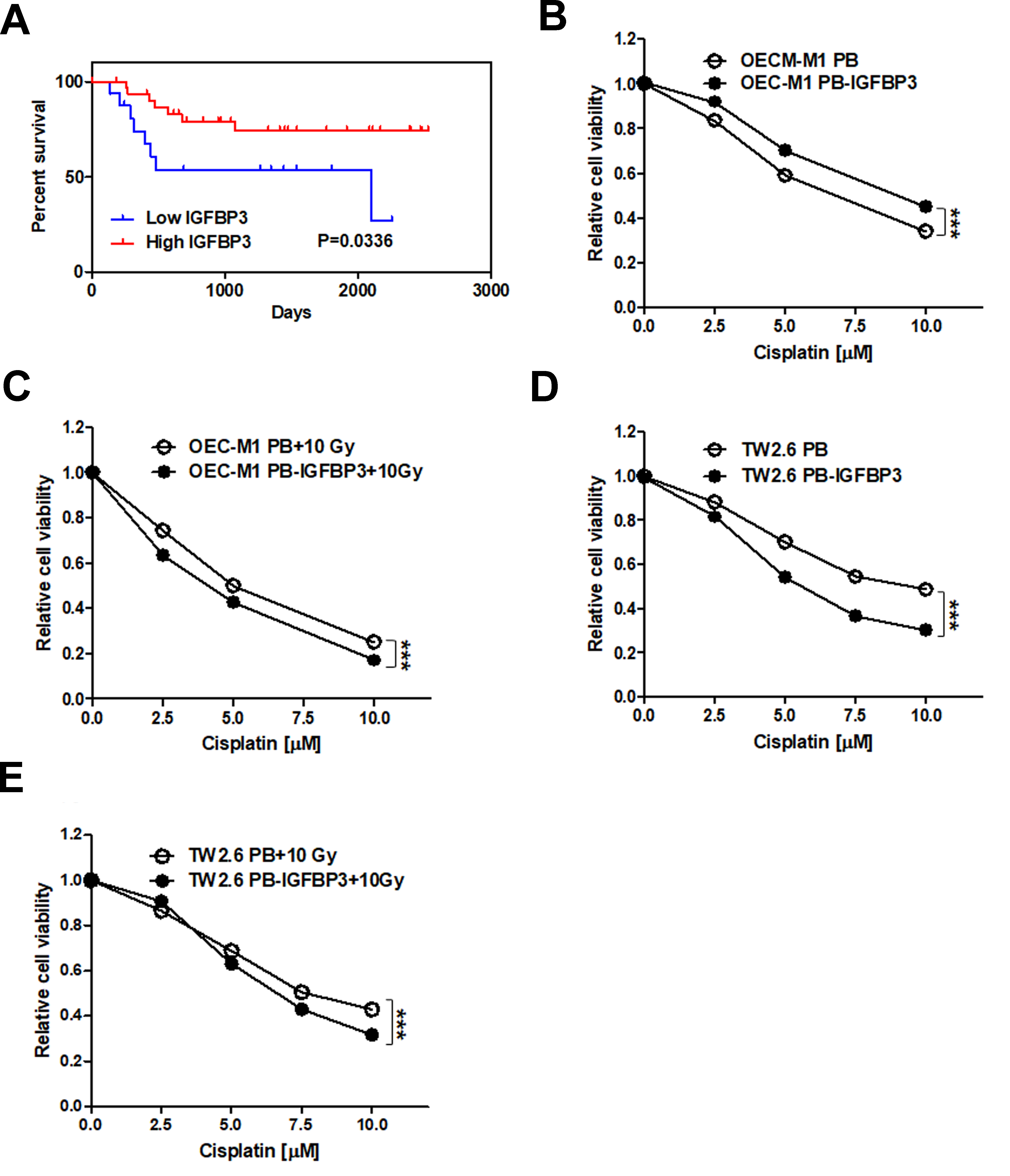

Supplement: Supplementary file 1 — Additional file 1: Figure S1. IGFBP3 enhanced radiation-induced cell-killing. (A) An overall survival correlation analysis was performed for 48 late-stage OSCC patient samples expressing different levels of IGFBP3. Patients were stratified into low (no or weak staining) and high (moderate or strong staining) groups based on IGFBP3 expression level; the log rank test was applied to detect significant differences in survival between groups. (B) Effects of cisplatin treatment on survival of OEC-M1 and (D) TW2.6 cells with ectopic IGFBP3 expression (PB-IGFBP3) versus vector controls (PB). Cells were treated with 0, 2.5, 5, 7.5, or 10 μM cisplatin and (C and E) combinations of 0, 2.5, 5, 7.5, or 10 μM cisplatin with 10 Gy IR and assessed for viability evaluated using the MTS assay at 72 hr after treatment. Results from one of at least two independent experiments are shown. Values are expressed in mean ± SE. *p<0.05; **p<0.01; ***p<0.001. [file 13046_2021_1898_MOESM1_ESM.tif]

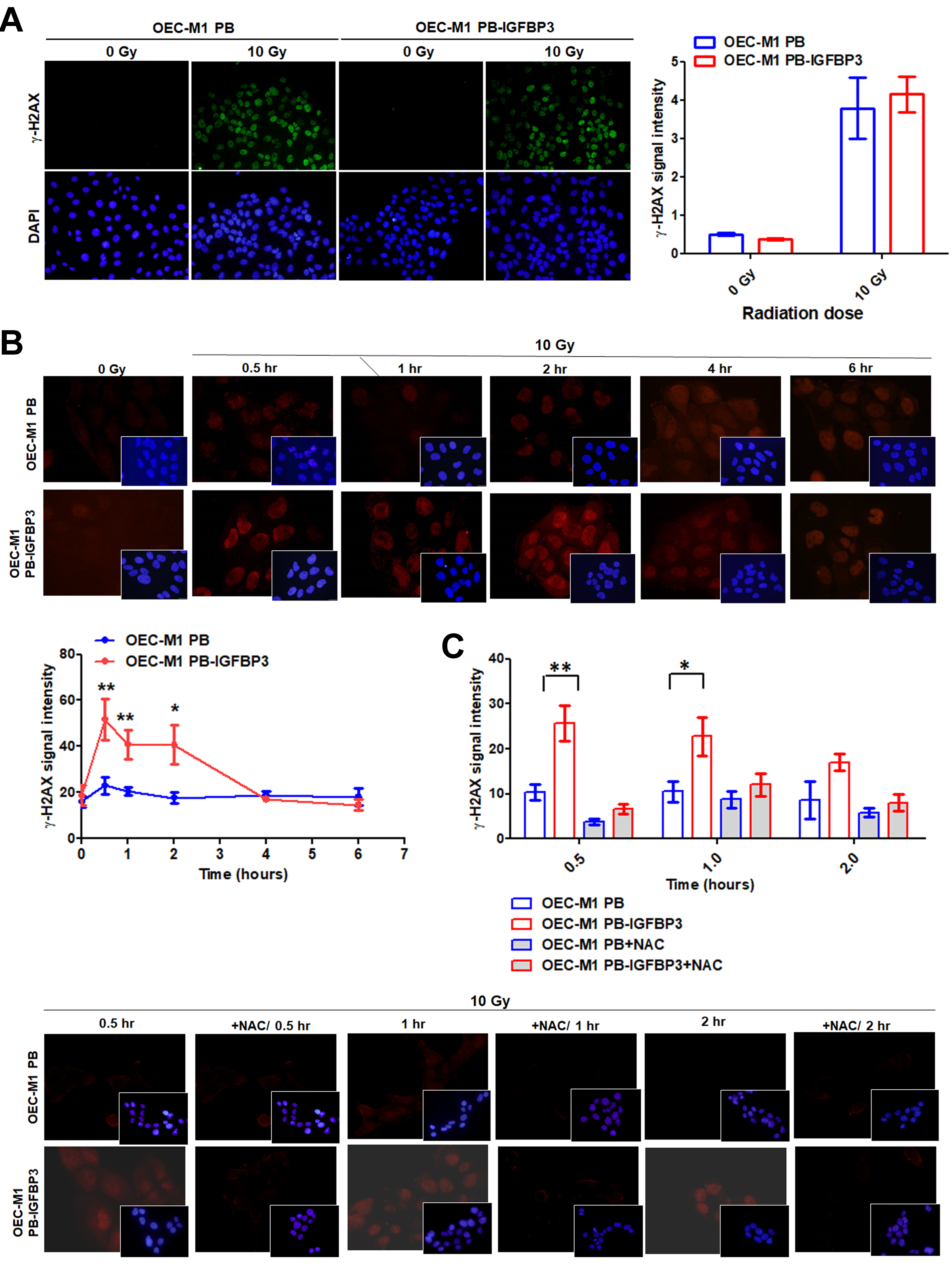

Supplement: Supplementary file 2 — Additional file 2: Figure S2. Ectopic IGFBP3 expression increased γ-H2AX in the early post-irradiation. (A) The incidence of γ-H2AX foci at 72 hr after 10 Gy IR exposure is shown. Left: Representative images of γ-H2AX staining of IGFBP3- and vector-expressing OEC-M1 cells irradiated with 10 Gy IR at 400× magnification. Green, γ-H2AX; blue, DNA stained with DAPI. Right: The mean signal intensity of γ-H2AX foci per cell. (B) The incidence of γ-H2AX foci in IGFBP3- and vector-expressing OEC-M1 cells at 0.5, 1, 2, 4 and 6 hr after 10 Gy IR exposure is shown. Upper: Representative images of γ-H2AX staining of IGFBP3- and vector-expressing OEC-M1 cells after 10 Gy IR irradiation at 200× magnification. Red, γ-H2AX; blue, DNA stained with DAPI. Lower: The mean signal intensity of γ-H2AX foci per cell at different time points post-irradiation. (C) The incidence of γ-H2AX foci in IGFBP3- and vector-expressing OEC-M1 cells treated with or without NAC at 0.5, 1 and 2 hr after 10 Gy IR exposure is shown. Lower: Representative images of γ-H2AX staining of IGFBP3- and vector-expressing OEC-M1 cells treated with NAC and 10 Gy IR at 200× magnification. Red, γ-H2AX; blue, DNA stained with DAPI. Upper: The mean signal intensity of γ-H2AX foci per cell at different time points post-irradiation. Results from one of at least two independent experiments are shown. Values are expressed in mean ± SE. *p<0.05; **p<0.01. [file 13046_2021_1898_MOESM2_ESM.tif]

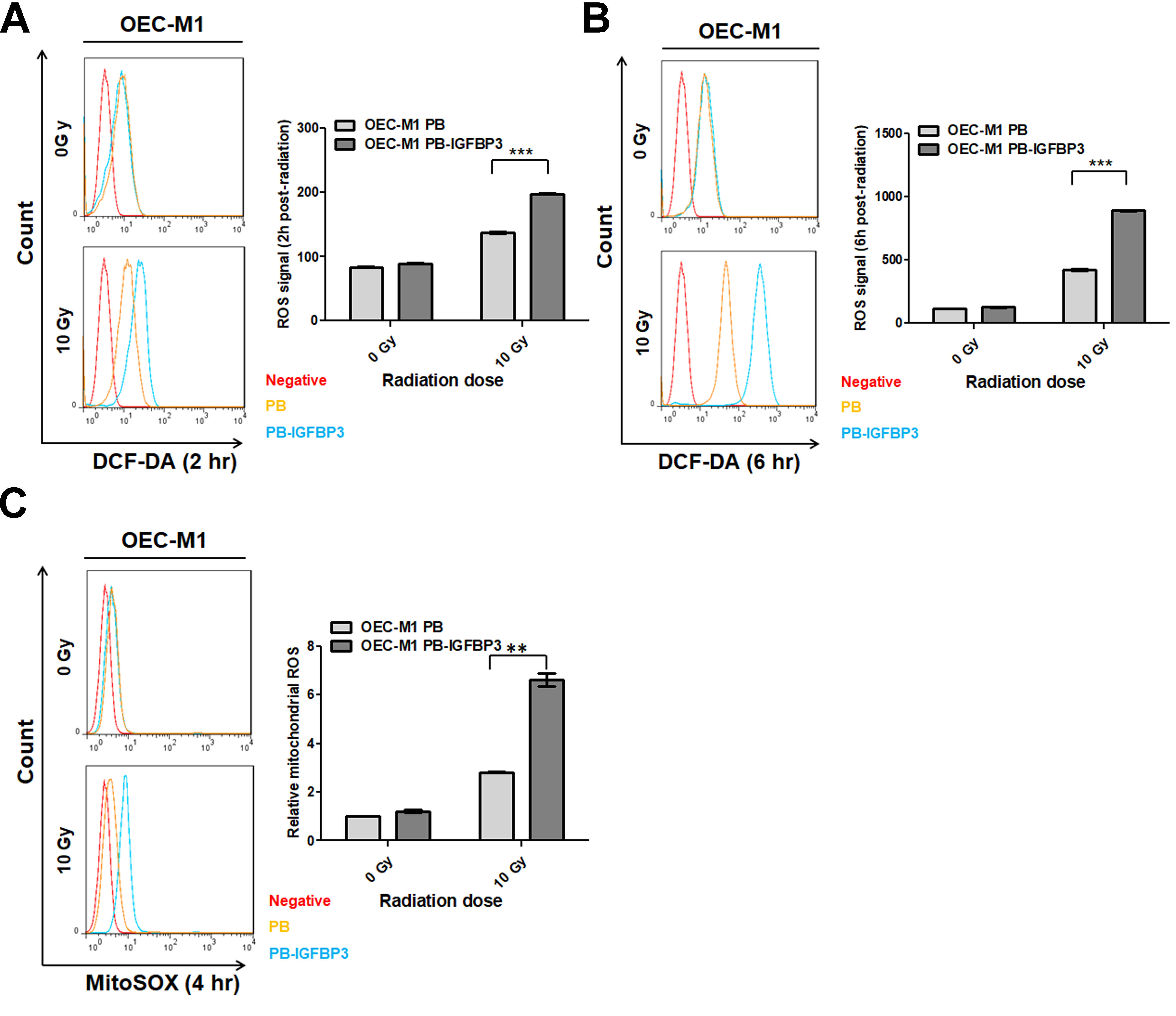

Supplement: Supplementary file 3 — Additional file 3: Figure S3. Ectopic IGFBP3 expression increased ROS production. (A) ROS production detected by DCF-DA at 2 hr and (B) 6 hr after 10 Gy IR in IGFBP3 expressing (PB-IGFBP3) or control (PB) OEC-M1 cells. Left: Representative diagrams of flow cytometric detection of ROS levels of each cell type (red line: negative control; orange line: vector control cells; blue line: IGFBP3 expressing cells). Right: Relative ROS signal from IGFBP3-expressing and vector control OEC-M1 cells with or without IR exposure. (C) Detection of mitochondrial ROS using MitoSOX at 4 hr post-irradiation with 10 Gy IR. Left: Representative diagrams of flow cytometric detection of ROS levels of each cell type (as described in part A above). Right: Relative mitochondrial ROS signal from IGFBP-expressing and vector control OEC-M1 cells with or without IR exposure. Mitochondrial ROS signal was determined by dividing the normalized levels in irradiated IGFBP3-expressing cells or vector control cells by that in non-irradiated control cells. Results from one of at least two independent experiments are shown. Values are expressed in mean ± SE. **p<0.01; ***p<0.001. [file 13046_2021_1898_MOESM3_ESM.tif]

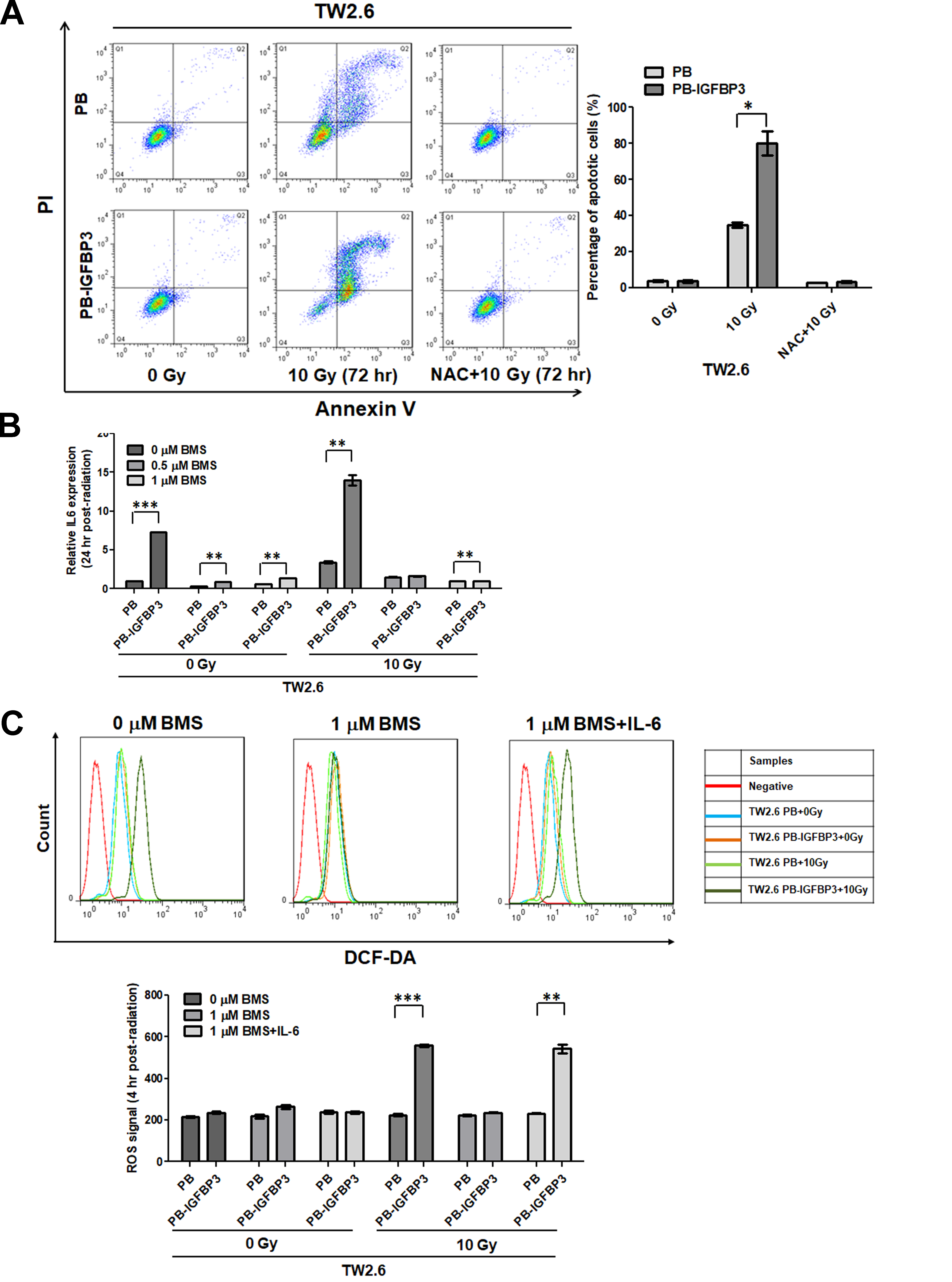

Supplement: Supplementary file 4 — Additional file 4: Figure S4. IGFBP3-mediated radiosensitivity in TW2.6 cells via the NF-κB/IL-6/ROS signaling axis. (A) Apoptosis assay using annexin V and propidium iodide (PI) in IGFBP3- and vector-expressing TW2.6 cells with or without IR exposure and NAC treatment. Left: A representative diagram of flow cytometric analysis with different quadrants indicating different stages of apoptosis (lower left quadrant: healthy; lower right: early apoptosis; upper right: late apoptosis). Right: Percentage of apoptotic cells following no irradiation or induction by 10 Gy IR with or without NAC treatment. (B) The IL6 level at 24 hr after exposure to 10 Gy IR in untreated or BMS-345541 (BMS)-treated TW2.6 cells expressing IGFBP3 (PB-IGFBP3) or the control vector (PB). All amplifications were normalized to β-actin. The relative mRNA expression in cells with ectopic IGFBP3 expression was normalized to that in untreated control cells. (C) Rescue of BMS-345541-suppressed ROS production by recombinant IL-6 in IGFBP3-expressing TW2.6 cells following irradiation. Upper: Representative diagrams of flow cytometric detection of ROS levels of each cell type with or without BMS-345541 or BMS plus IL-6 (red line, no cells; blue, non-irradiated vector expressing cells; orange, non-irradiated IGFBP3 expressing cells; green, irradiated vector control cells; dark green, irradiated IGFBP3 expressing cells). Lower: Relative ROS signal from IGFBP3-expressing (PB-IGFBP3) and vector control (PB) TW2.6 cells at 4 hr after irradiation with or without 10 Gy IR with or without BMS-345541 or BMS plus IL-6. Results from one of at least two independent experiments are shown. Values are expressed in mean ± SE. **p<0.01; ***p<0.001. [file 13046_2021_1898_MOESM4_ESM.tif]

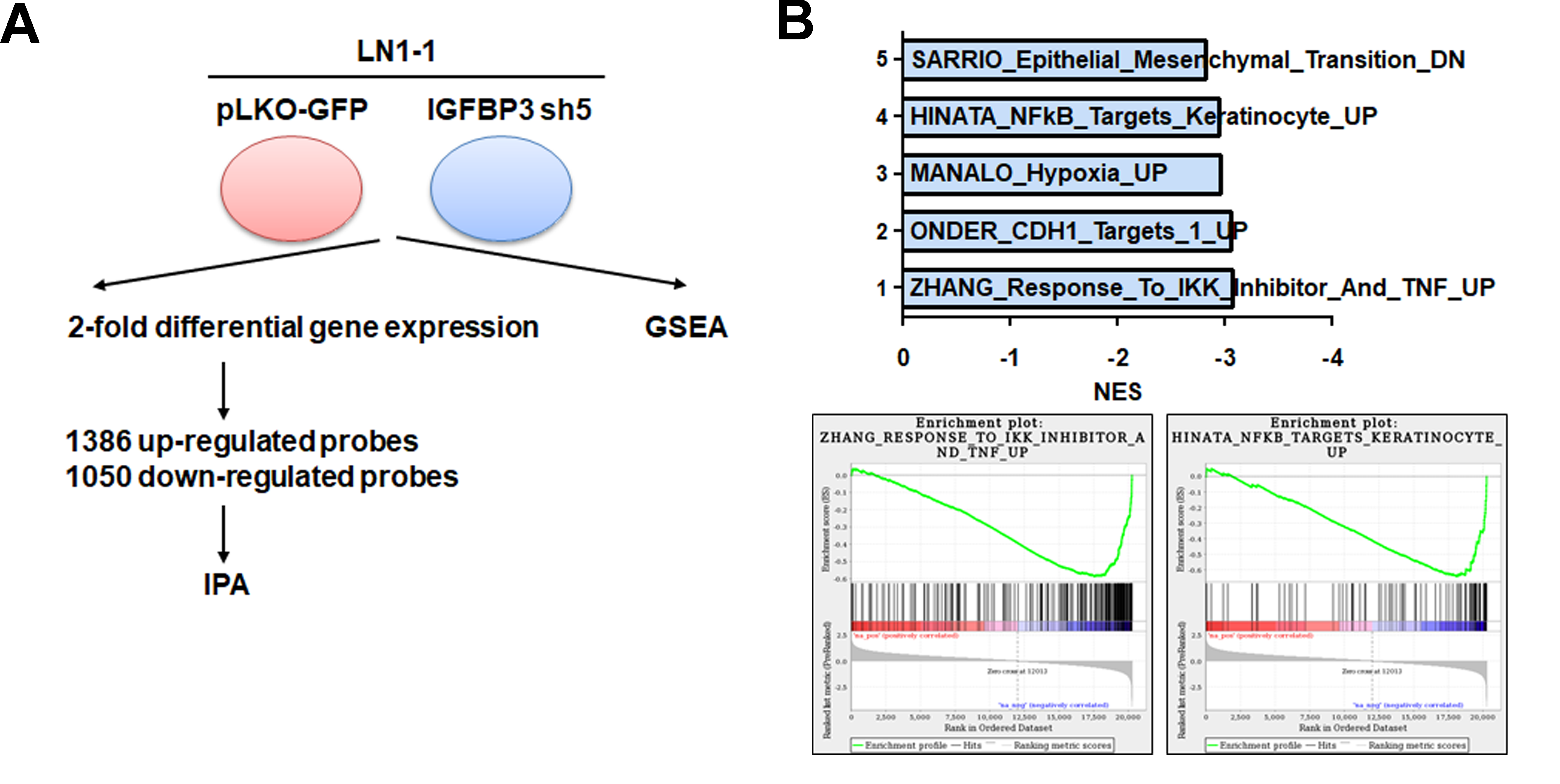

Supplement: Supplementary file 5 — Additional file 5: Figure S5. IGFBP3 knockdown inhibited NF-κB-related pathways. (A) The schematic for comparison of IGFBP3 knockdown cells with vector control cells via analysis using GSEA and IPA. (B) Upper: The most enriched datasets associated with IGFBP3 knockdown in LN1-1 cells by GSEA using C2 curated data sets. Light blue represents a negative normalized enrichment score (NES). Lower left: Enrichment plots of gene signatures of “ZHANG Response To IKK Inhibitor and TNF Up”. Lower right: Enrichment plots of gene signatures of “HINATA NF-κB Targets Keratinocyte Up”. [file 13046_2021_1898_MOESM5_ESM.tif]

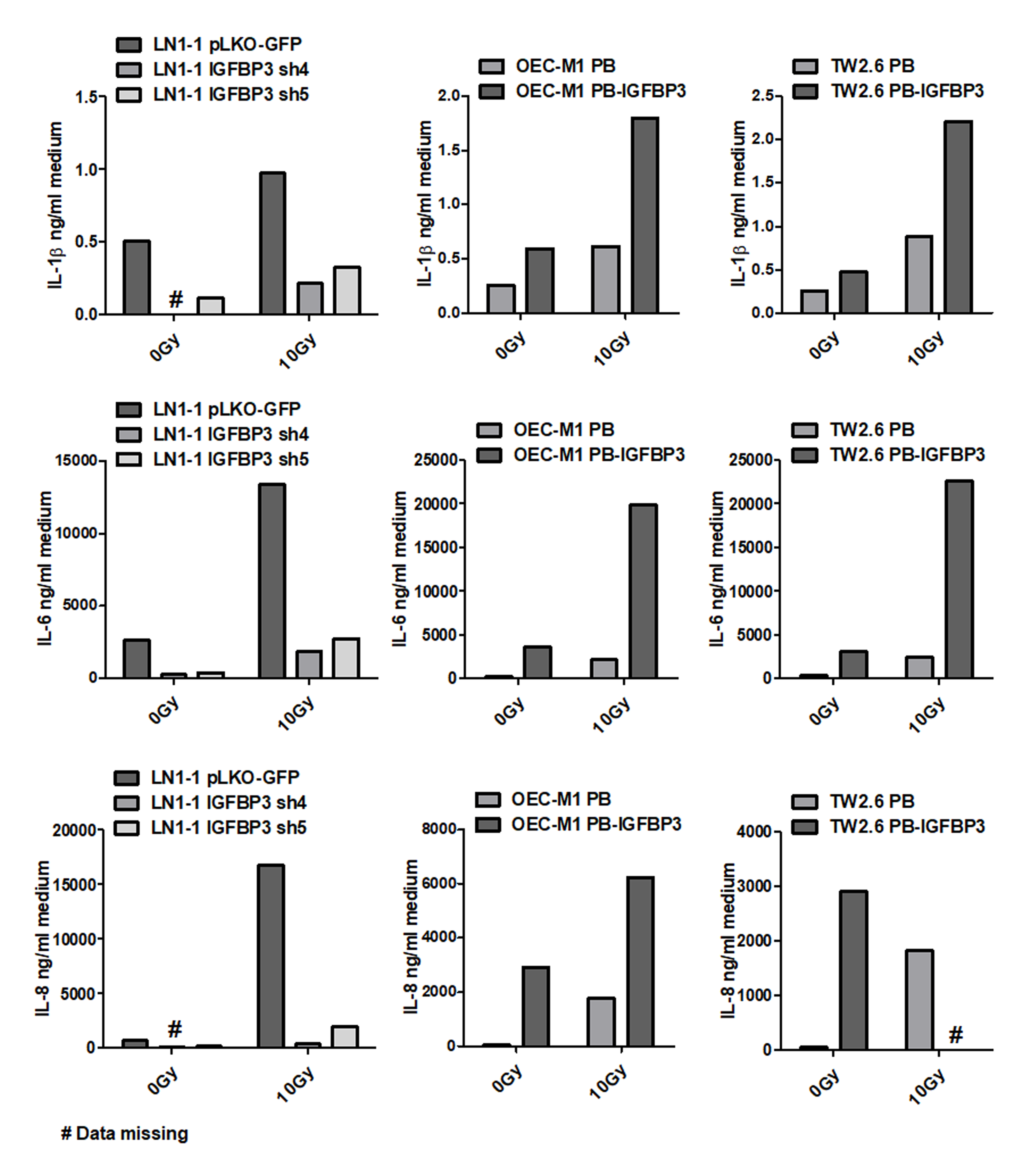

Supplement: Supplementary file 6 — Additional file 6: Figure S6. Levels of IL-1β, IL-6 and IL-8 in conditioned medium from IGFBP3 knockdown LN1-1, IGFBP3-expressing OEC-M1, and IGFBP3-expressing TW2.6 cells and their corresponding control cells at 24 hr after irradiation with 10 Gy IR as detected using the Bio-Plex assay. [file 13046_2021_1898_MOESM6_ESM.tif]
